# Transcriptome profiling provides new insights into the formation of floral scent in *Hedychium coronarium*

**DOI:** 10.1186/s12864-015-1653-7

**Published:** 2015-06-19

**Authors:** Yuechong Yue, Rangcai Yu, Yanping Fan

**Affiliations:** The Research Center for Ornamental Plants, College of Forestry and Landscape Architecture, South China Agricultural University, Guangzhou, 510642 China; College of Life Sciences, South China Agricultural University, Guangzhou, 510642 China

**Keywords:** *Hedychium coronarium*, Zingiberaceae, Transcriptome, Floral scent, Secondary metabolism, Terpenoid, Benzenoid, Transcription factor

## Abstract

**Background:**

*Hedychium coronarium* is a popular ornamental plant in tropical and subtropical regions because its flowers not only possess intense and inviting fragrance but also enjoy elegant shape. The fragrance results from volatile terpenes and benzenoids presented in the floral scent profile. However, in this species, even in monocots, little is known about the underlying molecular mechanism of floral scent production.

**Results:**

Using Illumina platform, approximately 81 million high-quality reads were obtained from a pooled cDNA library. The *de novo* assembly resulted in a transcriptome with 65,591 unigenes, 50.90 % of which were annotated using public databases. Digital gene expression (DGE) profiling analysis revealed 7,796 differential expression genes (DEGs) during petal development. GO term classification and KEGG pathway analysis indicated that the levels of transcripts changed significantly in “metabolic process”, including “terpenoid biosynthetic process”. Through a systematic analysis, 35 and 33 candidate genes might be involved in the biosynthesis of floral volatile terpenes and benzenoids, respectively. Among them, flower-specific HcDXS2A, HcGPPS, HcTPSs, HcCNL and HcBCMT1 might play critical roles in regulating the formation of floral fragrance through DGE profiling coupled with floral volatile profiling analyses. In vitro characterization showed that HcTPS6 was capable of generating β-farnesene as its main product. In the transcriptome, 1,741 transcription factors (TFs) were identified and 474 TFs showed differential expression during petal development. It is supposed that two R2R3-MYBs with flower-specific and developmental expression might be involved in the scent production.

**Conclusions:**

The novel transcriptome and DGE profiling provide an important resource for functional genomics studies and give us a dynamic view of biological process during petal development in *H. coronarium*. These data lay the basis for elucidating the molecular mechanism of floral scent formation and regulation in monocot. The results also provide the opportunities for genetic modification of floral scent profile in *Hedychium*.

**Electronic supplementary material:**

The online version of this article (doi:10.1186/s12864-015-1653-7) contains supplementary material, which is available to authorized users.

## Background

Floral scent has become as one of the most important traits for ornamental plants. Many consumers prefer scented flowers and are willing to pay extra to get them [1]. The components of floral scent have been widely applied to the perfumes, cosmetics, flavourings and medicinal preparations [2]. To the plant itself, the primary function of floral scent is to attract and guide pollinators to ensure reproductive success [3]. In addition, the anti-microbial or anti-herbivore activity of compounds emitted from flowers protects the vulnerable reproductive organs of plants from their enemies [4]. Likewise, floral volatiles also function in the defense of abiotic stresses such as high light, temperature or oxidative stress [5]. Floral scent is determined by a complex mixture of low-molecular weight lipophilic molecules with low boiling points and high vapor pressures at ambient temperature [6]. Up to now, more than 1,700 chemical compounds have been identified from the floral scent profiles of over 1,000 seed plants [7]. These compounds mainly fell into the terpenoid and benzenoid/phenylpropanoid classes.

Terpenoids represent the largest and most diverse group of floral scent volatiles, which are derived from two common isoprene precursors, isopentenyl pyrophosphate (IPP) and its allylic isomer dimethylallyl pyrophosphate (DMAPP) [5]. In plants, the isomer precursors are synthesized via two alternative and independent pathways: the cytosolic mevalonate (MVA) and the plastidial 2-C-methyl-D-erythritol 4-phosphate (MEP) pathways. Thereafter, the successive head-to-tail condensation of IPP and DMAPP by the action of prenyltransferases generates the direct precursors of terpenes, geranyl diphosphate (GPP), geranylgeranyl diphosphate (GGPP) in plastids, and farnesyl diphosphate (FPP) in cytosol or mitochondria [8]. In the last step, plastidial terpene synthases (TPSs) convert the GPP and GGPP into diverse monoterpenes and diterpenes, while cytosolic/mitochondrial TPSs catalyze the FPP into various sesquiterpenes [9]. Previous researches on the biosynthesis of floral volatile terpenes predominantly focused on the isolation and characterization of TPSs. After the first enzyme involved in the formation of floral scent, *S*-linalool synthase, was purified and characterized in the flowers of *Clarkia breweri* [10, 11], some TPSs responsible for the biosynthesis of floral volatile terpenes in model plant Arabidopsis [12, 13], and some ornamental plants rose [14], snapdragon [15, 16] and *Nicotiana* [17] have been investigated. Most of the above *TPSs* transcript exhibit flower-specific, developmental and rhythmic expression. Nevertheless, there is limited information regarding the transcriptional regulation mechanism of the floral volatile terpenes biosynthesis. Until now, MYC2, a basic helix-loop-helix (bHLH) TF in Arabidopsis, is the only identified TF that could directly regulate the formation of floral volatile terpenes [18]. By mediating gibberellic acid and jasmonic acid signals, MYC2 directly binds to the promoters of two sesquiterpene synthase genes *TPS11* and *TPS21* which account for the floral sesquiterpenes production, and activating their expression [18]. Recently, overexpression of Arabidopsis *PAP1* TF in roses resulted in the enhanced production of phenylpropanoid and terpenoid scent compounds [19]. However, the regulatory mechanism that this TF mediated in transgenic rose remains unclear.

Benzenoids/phenylpropanoids are the second largest class of floral scent and initiate from the aromatic amino acid phenylalanine, which is generated via shikimate pathway and arogenate pathway in plastids [20]. After phenylalanine was exported from plastids, phenylalanine ammonialyase (PAL) catalyzes phenylalanine into cinnamic acid and represents the first committed step in benzenoids/phenylpropanoids biosynthesis [21]. The volatile phenylpropenes, such as eugenol and isoeugenol, share the initial biosynthetic steps with lignin and then undergo two enzymatic reactions to eliminate the oxygen functionality at C9 position of coniferyl alcohol [5]. On the other hand, the conversion of cinnamic acid to volatile benzenoids requires the cleavage of two carbons from the propyl side-chain and has been reported to proceed via β-oxidative pathway and non β-oxidative pathway [21, 22]. The β-oxidative pathway has recently been elucidated in petunia flowers [23–26] and Arabidopsis seeds [27], including cinnamic acid import, activation of cinnamic acid to its CoA thioester, hydration, oxidation, and thiolysis in peroxisome. The non-β-oxidative pathway remains incomplete and the enzymes resulting in the formation of benzaldehyde are still unknown. Only the last enzymatic reaction, oxidation of benzaldehyde into benzoic acid, was described in snapdragon [28]. In the final step, two enzyme superfamilies, the SABATH family of methyltransferases and BAHD superfamily of acyltransferases, are considered to play critical roles in the biosynthesis of volatile benzenoids [5]. They are well investigated in *Clarkia breweri*, petunia and snapdragon. Several TFs regulating the expression of genes encoding enzymes involved in benzenoids/phenylpropanoids production have been identified in petunia. ODO1, a member of R2R3-type MYB family, is the first TF characterized as the regulator of scent production in flowers and regulates the expression level of several structural genes in shikimate pathway [29]. Meanwhile, ODO1 is directly regulated by another flower-specific R2R3-type MYB TF, EOBII, which also controls several genes encoding enzymes involved in the benzenoid/phenylpropanoid pathway and activates the promoter of the isoeugenol synthase gene [30, 31]. Recently, a new flower-specific R2R3-type TF, EOBI, was identified in petunia, which acts downstream of EOBII and also regulates ODO1 and scent-related functional genes [32]. In addition, petunia PhMYB4 fine-tunes the floral scent by repressing the transcription of cinnamate-4-hydroxylase gene, thus leading to the flux towards volatile phenylpropanoids [33].

Expressed sequence tag (EST) sequencing constitutes a useful approach for gene discovery in the last decades. However, this approach has some limitations, such as relatively low throughput, high cost and lack of gene quantification [34]. Transcriptome sequencing (RNA-Seq) based on next-generation sequencing technology is a cost-effective and highly efficient approach to transcriptome profiling [34]. Recent advances in RNA-Seq make it act as a popular and powerful tool for large scale sequencing in the non-model plant without a reference genome [35]. DGE profiling provides a precise measurement of transcripts levels in different samples and allows the intercomparison among samples to identify DEGs. RNA-Seq coupled with DGE profiling is very helpful for identifying candidate genes accounted for the biosynthesis of secondary metabolites in non-model plant. Base on this approach, a number of secondary metabolism pathways were investigated in plants with little genomic information, such as in *Taxus* [36], *Siraitia grosvenorii* [37], *Momordica cochinchinensis* [38] and grape hyacinth [39].

*H. coronarium* (commonly known as butterfly ginger or white ginger lily, a member of the Zingiberaceae family) originates from the Himalaya region and southern China. Nowadays, it is cultivated as an ornamental plant in many tropical and subtropical regions. In addition, essential oils of its flowers and rhizomes are used in perfumery while rhizomes and stems are applied to pharmaceutical preparations due to its diuretic, antidiabetic, antisyphilitic and anti-inflammatory properties [40–42]. Despite the high ornamental and medicinal values of *H. coronarium*, there is no genomic information available to this species. When blooming, the flowers of *H. coronarium* are intensely fragranced and produce a mixture of floral volatiles including monoterpenes β-ocimene, linalool, 1,8-cineole, sesquiterpene α-farnesene and benzenoids methyl benzoate. Previous researches mainly focused on composition analyses of floral scent and essential oil [43, 44]. The molecular mechanisms of biosynthesis and regulation of these compounds in this species are less well understood. To date, only one FPPS and two TPSs were identified and characterized in *H. coronarium* [45, 46]. Meanwhile, much more attention in previous researches on floral scent at molecular level paid to dicots than to monocots. In addition, many species in *Hedychium* possess high ornamental values for their showy flowers with various colors and shapes. However, some species with fine ornamental traits in *Hedychium* lack the fragrance, such as *H. coccineum* [40]. Therefore, researches on the formation of floral scent in scented *H. coronarium* will facilitate the breeding of scent-related traits in *Hedychium*.

In this study, a cDNA library including equal amount of RNA taken from flowers at three developmental stages, leaves and rhizomes in *H. coronarium* were deeply sequenced on Illumina platform. The *de novo* assembly of more than 81 M high-quality reads resulted in a *H. coronarium* transcriptome with 65,591 unigenes. Moreover, three DGE libraries were generated to compare gene expression patterns during the petal development. Furthermore, the emissions of floral volatile compounds and the expression patterns of genes involved in the floral scent formation were analyzed. The activity of HcTPS6 was characterized through biochemical methods. Finally, floral scent-related TFs were analyzed and discussed.

## Results

### Changes of volatile compounds during flower development

**Fig. 1:**
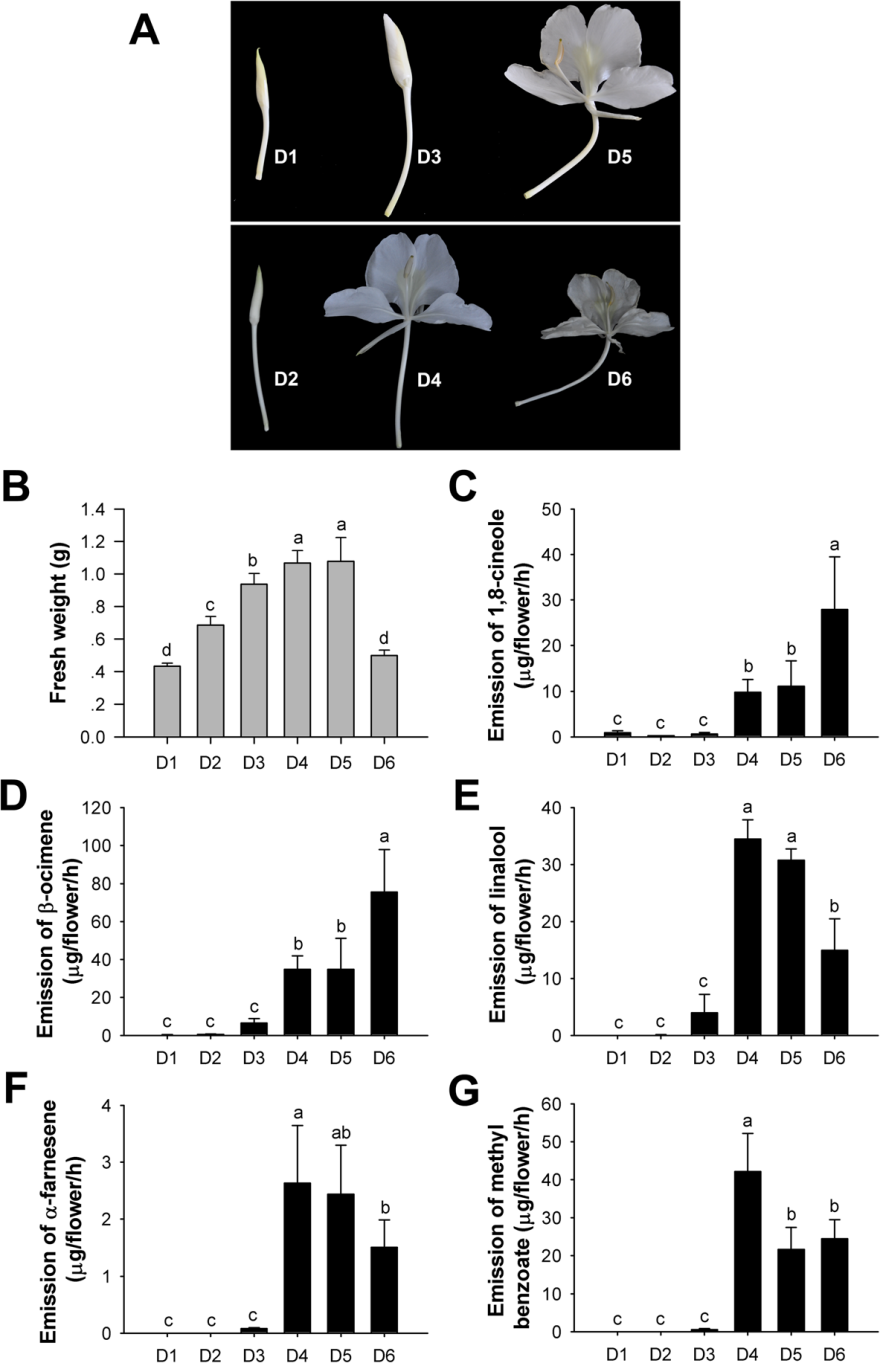
Changes of the main volatile compounds during flower development in *H. coronarium*. **a** Photographs of flowers at different flower developmental stages. **b** Change of fresh weight in flower. Data are means ± SD (*n* = 6). **c**-**g** Emission of 1,8-cineole (**c**), β-ocimene (**d**), linalool (**e**), α-farnesene (**f**) and methyl benzoate (**g**) in flowers at different stages of development. Data are means ± SD (*n* = 3). Different lowercase letters labeled on bars indicate statistically significant differences at the level of *P* < 0.05

To investigate the formation and regulation of floral scent during flower development, the flower developmental process from squaring stage to senescence stage was divided into six developmental stages with 12-h intervals (designated D1 to D6) (Fig. 1a). The fresh weight of individual flower gradually increased along with the flower opening, reaching the highest level at blooming period (D4-D5), while the fresh weight declined by 53.7 % in the process of senescence (D5-D6) (Fig. 1b). Volatile compounds emitted from flowers at six stages were sampled by headspace collection and detected by gas chromatograph-mass spectrometer (GC-MS). At the blooming stage (D4), the floral scent profile predominantly consisted of the monoterpene linalool, β-ocimene, 1,8-cineole and sesquiterpene α-farnesene as well as methyl benzoate (Fig. 1c-g). Methyl benzoate, β-ocimene and linalool were dominated in the floral scent profile. The emission of 1,8-cineole and β-ocimene was low at bud period (D1-D3) and increased strongly when anthesis (D4), while the emissions continuously increased by 2.5 and 2.2 times in the process of senescence, respectively (Fig. 1c and d). Linalool, α-farnesene and methyl benzoate were hardly detectable at D1 and D2 stages. Emission of these volatiles peaked at D4 and declined gradually thereafter (Fig. 1e-g). These results revealed all the volatile compounds mentioned above were controlled developmentally.

### Sequencing and *de novo* assembly

For no genomic information in *H. coronarium* was available, the transcriptome sequencing and assembly was first performed. To obtain a comprehensive *H. coronarium* transcriptome, a pooled cDNA library, which is synthesized from equal quantities of RNA isolated from flowers at D1, D4 and D6 stages as well as leaves and rhizomes, was paired-end sequenced on an Illumina Hiseq 2000 platform. Approximately 84.29 million raw reads were generated. After removal of adaptor sequences, ambiguous nucleotides and low-quality sequences, assembly of 81.59 million high-quality clean reads resulted in 65,591 unigenes. An overview of the sequencing and assembly is outlined in Table 1. The N50 value [47], widely used to assess the quality of sequence assembly, was 1,284 bp, suggesting a better assembly. The assembly generated a number of larger unigenes: 5,154 unigenes longer than 2,000 bp, 8,358 unigenes between 1,001-2000 bp, and 11,899 unigenes between 501–1000 bp (Additional file 1).

**Table 1:** Sumamary of the *H. coronarium* transcriptome

Total number of raw reads	84,289,070
Total number of clean reads	81,594,084
Total Clean base pairs (Gbp)	8.08
GC content (%)	48.57
Error rate (%)	0.03
Q20 (%)	97.67
Total number of unigenes	65,591
Mean length of unigenes (bp)	732
Min length of unigenes (bp)	201
Max length of unigenes (bp)	15,670
N50 (bp)	1,284

### Functional annotation and classification

**Fig. 2:**
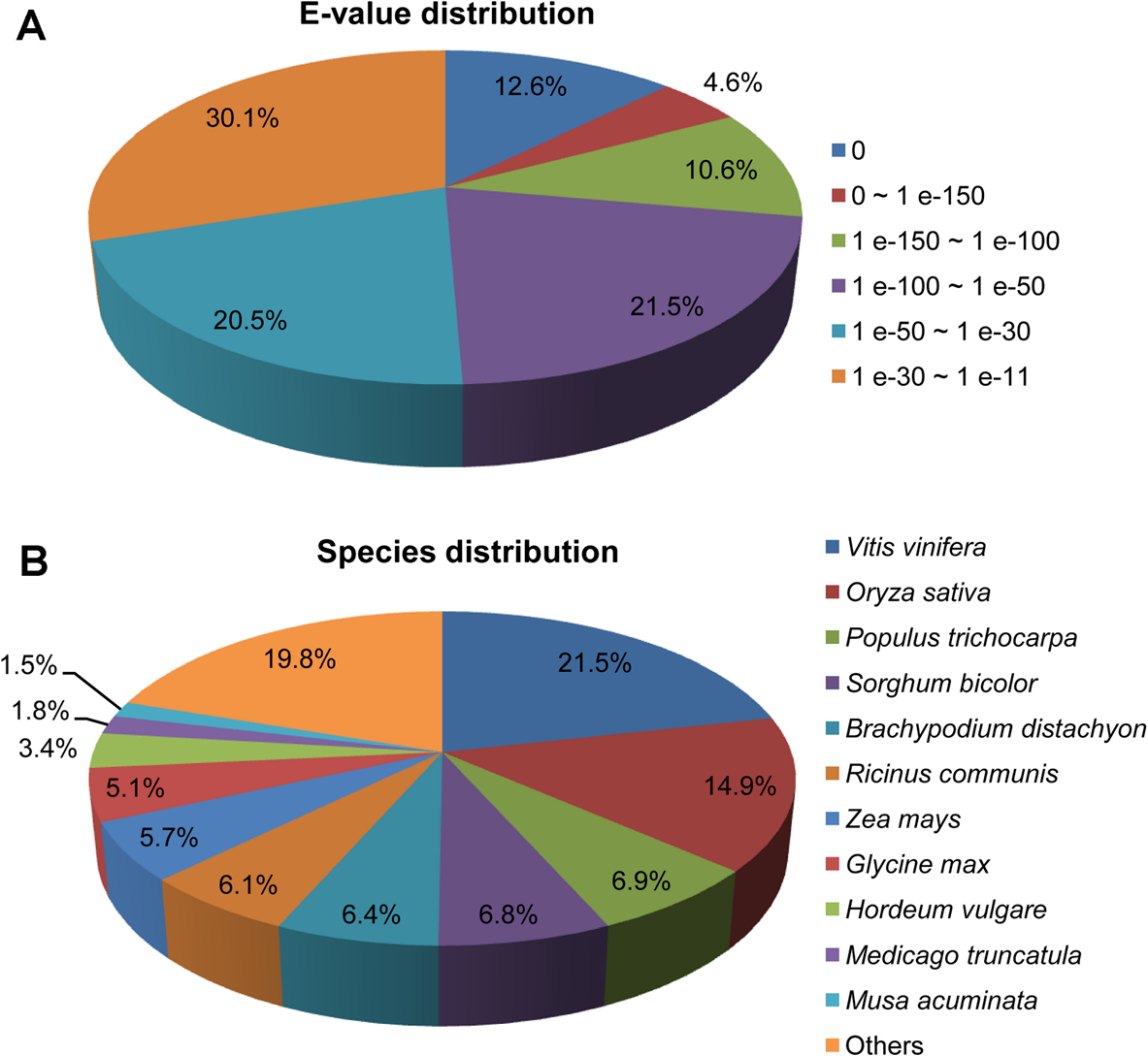
Characteristics of homology search of unigenes against the Nr database. **a** E-value and (**b**) species distribution of the top BLAST hits for each unique sequence. The cut-off values for BLAST search was set at 1.0e^−10^

In order to maximize the information of novel assembled unigenes, all unigene sequences were searched against seven public databases: NCBI non-redundant protein (Nr) database, NCBI non-redundant nucleotide (Nt) database, Swiss-Prot protein database, Protein family (Pfam), Gene Ontology (GO), euKaryotic Ortholog Groups (KOG) and Kyoto Encyclopedia of Genes and Genomes (KEGG) database. 33,389 distinct sequences were annotated using this strategy, accounting for 50.90 % of the total unigenes (Table 2). Among 32,202 unannotated unigenes, 26,942 (83.67 %) were less than 500 bp (data not shown), indicating the importance of unigenes length for gene annotation. Based on BLASTX search against the Nr database with an *E*-value cut-off of 10^−10^, 26,302 unigenes (40.10 % of all unigenes) returned a significant BLAST hits (Table 2). Among them, 12.62 % of the annotated sequences had very strong homology (*E*-value = 0) to the top matched sequences, and 36.72 % showed strong homology (0 < *E*-value < 10^−50^), while 50.65 % of the homologous sequences ranged between 10^−50^ and 10^−11^ (Fig. 2a). With respect to species, 21.46 % of the unigenes have top hits to genes from *Vitis vinifera*, followed by *Oryza sativa* (14.93 %), *Populus trichocarpa* (6.95 %), *Sorghum bicolor* (6.80 %) and *Brachypodium distachyon* (6.37 %) (Fig. 2b).

**Table 2:** Summary of annotations on unigenes against public databases

Database	Number of annotated unigene	Percent of annotated unigenes (%)
Nr	26,302	40.10
Nt	9,477	14.45
Swiss-prot	21,118	32.20
Pfam	22,998	35.06
GO	24,232	36.95
KOG	13,240	20.19
KO	7,923	12.08
Total	33,389	50.90

To functional categorize *H. coronarium* transcriptome unigenes, GO assignments were performed using Blast2GO. Based on sequence homology, 24,232 (36.95 %) unigenes were classified into three major functional categories (biological process, cellular component and molecular function) and 60 subcategories (Table 2 and Additional file 2). In the biological process category, dominant subcategories were “metabolic process” and “cellular process”. Among cellular component terms, “cell” and “cell part” were observed to be the most abundant classes. Subcategory “binding” and “catalytic activity” showed a high percentage of unigenes in the category of molecular function. To classify orthologous proteins, the assembled unigenes were compared against KOG, which presents in the Cluster of Orthologous Groups (COG) database and contains protein sequences from seven eukaryotic genomes [48]. 13,240 unigenes were grouped into 26 KOG classifications (Table 2 and Additional file 3). The cluster “general function prediction only” represented the largest category, followed by “post-translational modification, protein turnover, chaperones”, “signal transduction”, “transcription”, “cytoskeleton” and “transcription”. In particular, the category of “secondary metabolites biosynthesis, transport and catabolism” with 477 unigenes were focused on, because of the importance of secondary metabolites to floral scent and color as well as medicinal components. To identify the biological pathways that are related to unigenes, the unigenes sequences were annotated with KEGG orthology (KO), and then mapped to the reference canonical pathways in KEGG [49]. Totally, 7,923 unigenes were annotated and 5,933 sequences were assigned to 119 KEGG pathways (Table 2 and Additional file 4). “Metabolism” and “Genetic information processing” were dominant in primary pathway hierarchy. In secondary pathway hierarchy, “carbohydrate metabolism” and “translation” represented the most abundant classes. The pathways with most representation by the unigenes sequences were “ribosome” (584 members), followed by “oxidative phosphorylation” (325 members), “protein processing in endoplasmic reticulum” (260 members) and “plant hormone signal transduction” (232 members). With respect to the pathways related to secondary metabolism, “phenylpropanoid biosynthesis” (109 members) represented the largest group, followed by “terpenoid backbone biosynthesis” (80 members), “flavonoid biosynthesis” (43 members) and “carotenoid biosynthesis” (35 members) (Additional file 5). All these analyses upon the sequences generated in this study provide a valuable resource for gene discovery and gene functional research in specific biological event in *H. coronarium*.

### DGE sequencing and gene quantification

Given that petals are the main tissue for floral volatile compounds emission in *H. coronarium* [46], and the release amounts of these compounds at squaring stage (D1), blooming stage (D4) and senescence stage (D6) display significant difference (Fig. 1), petals at these stages were chosen for DGE profiling analysis. After three DGE libraries were sequenced on Illumina platform, approximately 9.30, 7.94 and 7.53 million raw reads were generated, respectively. After filtering the raw data, approximately 9.16, 7.84 and 7.43 million clean reads were mapped to *H. coronarium* reference transcriptome. The mapped reads in three libraries accounted for 92.33 %, 94.21 % and 94.13 % of clean reads, respectively, indicating an ideal DGE sequencing and mapping. An overview of the sequencing and mapping is outlined in Table 3. The uniform distribution of reads on reference genes revealed no obvious biases for specific gene regions (Additional file 6), suggesting well sequencing randomness.

**Table 3:** Summary of DGE sequencing and mapping during petal development

Description	D1	D4	D6
Total raw reads	9,297,866	7,940,092	7,525,179
Total clean reads	9,156,081	7,837,458	7,428,674
Clean bases (G)	0.92	0.78	0.74
Q20 (%)	98.05	97.92	98.03
GC content (%)	47.56	47.76	47.25
Total mapped reads	8,453,498	7,383,651	6,992,976
Total mapped reads/Total clean reads (%)	92.33	94.21	94.13

To quantify the gene expression level, the number of mapped reads for each gene was calculated, and then normalized to reads per kilobase of exon model per million mapped reads (RPKM). The RPKM approach facilitates transparent comparison of gene expression levels within or between samples [50]. Genes with RPKM < 0.1 were defined as no transcription; Genes with RPKM in the interval 0.1-3.57, 3.57-15 and > 15 were considered to be expressed at low, medium and high levels (Table 4) [51]. The gene number at medium and high expression levels in D1 stage was larger than in D4 and D6, while the number of unexpressed genes in D1 stage was less than in D4 and D6 (Table 4), indicating more genes function in D1 stage. To check whether the sequencing depth allowed a reliable calculation of gene expression levels, the saturation curves analysis was performed. When RPKM was greater than 5, the curves reached saturation in principle before 100 % mapped reads were used. However, for RPKM intervals 1–5 and < 0.1, when 90 % mapped reads were used, the fraction of genes number with RPKM value within 15 % error of the final value only reached approximately 0.85 (Additional file 7). Therefore, we hold that the gene quantification was reliable in this experimental condition when the gene expression was at medium and high expression levels.

**Table 4:** Statistics of gene expression abundance in three petal developmental stages

RPKM interval	Expression level	D1	D4	D6
0-0.1	no	17,765 (27.08 %)	22,483 (34.28 %)	22,843 (34.83 %)
0.1-3.57	low	29, 146 (44.44 %)	29,935 (45.64 %)	28,720 (43.79 %)
3.57-15	medium	10,459 (15.95 %)	8,031 (12.24 %)	8,273 (12.61 %)
15-60	high	6, 176 (9.42 %)	3,822 (5.83 %)	4,182 (6.38 %)
>60	very high	2,045 (3.12 %)	1,320 (2.01 %)	1,573 (2.40 %)

### Analysis of DEGs during petal development

**Fig. 3:**
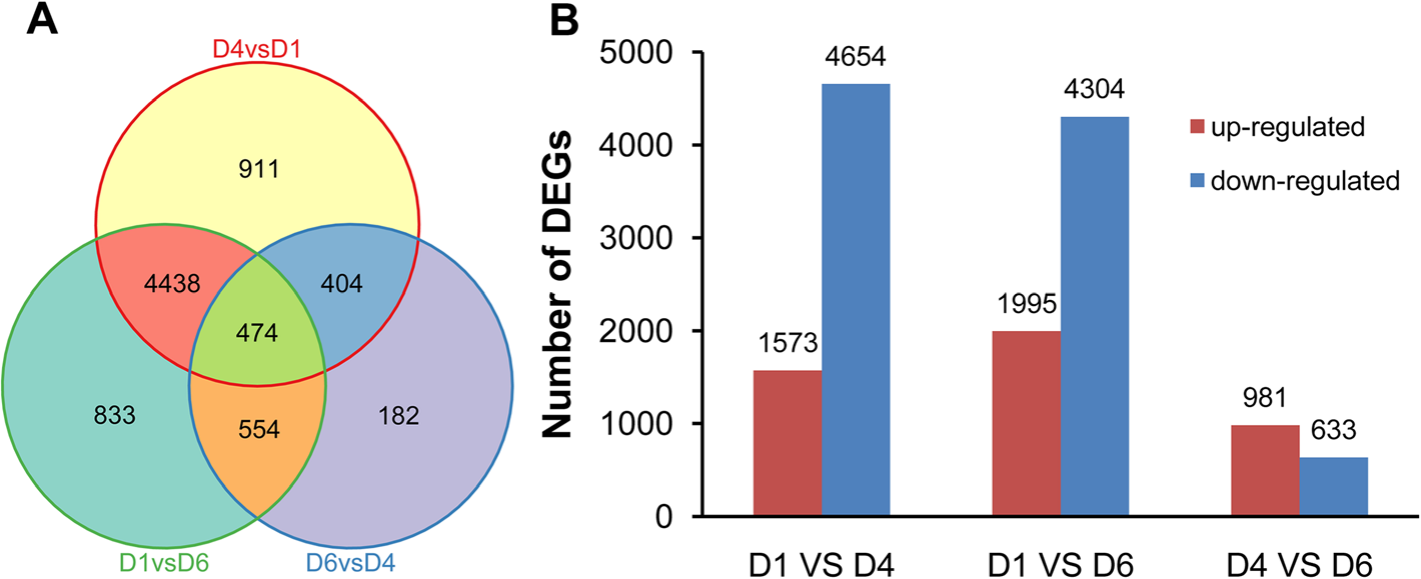
Gene expression comparisons. **a** Venn diagram of number of DEGs. Genes in overlapping sets show the differential expression in two or three comparison pairs. **b** Changes in gene expression profile. The numbers of up-regulated and down-regulated genes between D1 and D4, D1 and D6, D4 and D6 are summarized

To identify significant DEGs during petal development, the expression quantity of each gene in three petal libraries was compared pairwise and filtered with |log_2_(fold change)| > 1 and *q* value < 0.005. A total of 7,796 DEGs were detected among three libraries (Fig. 3), accounting for 11.89 % of total unigenes in *H. coronarium* transcriptome. Among them, 474 genes showed significantly differential expression in all three developmental stages; 4,438 genes in D1, 404 genes in D4 and 554 genes in D6 showed significantly differential expression with two other developmental stages (Fig. 3a). As shown in Fig. 3b, the down-regulated DEGs in D1 VS D4 and D1 VS D6 comparisons, were greater than in D4 VS D6 comparison, suggesting more complex biological event occurred in petals at squaring stage than at blooming and senescence stage.

To investigate the biological event that DEGs mainly involved during petal development, GO term enrichment analysis was conducted upon Wallenius non-central hyper-geometric distribution (Additional file 8). In the category of biological process, GO terms “metabolic process”, “oxidation-reduction process”, “protein folding”, “translation”, “proteolysis involved in cellular protein catabolic process”, “terpenoid biosynthetic process” and “steroid biosynthetic process” were significantly enriched in comparison of D1 and D4 stages. 77.66 % of total 367 members and 68.86 % of 533 total members in “metabolic process” and “oxidation-reduction process” were down-regulated, respectively. In the term “protein folding” and “translation”, there are 83 out of 90 genes and 168 out of 182 genes down-regulated, respectively. All 15 members in “proteolysis involved in cellular protein catabolic process” were down-regulated. However, between D4 and D6 stages, “proteolysis involved in cellular protein catabolic process” with all 15 members up-regulated was the only significantly enriched GO term. In the cellular component category, eleven GO terms were significantly enriched in D1 VS D4 comparison, while only three GO terms in D4 VS D6 comparison, indicating that more cellular component underwent active changes in the blooming process. In the category of molecular function, there were ten GO terms significantly enriched in D1 VS D4 comparison, including “oxidoreductase activity”, “catalytic activity”, “structural constituent of ribosome”, “coenzyme binding”, et al. Only “threonine-type endopeptidase activity” and “endopeptidase activity” were significantly enriched between D4 and D6 stages.

To identify pathways in which DEGs are significantly enriched during petal development, DEGs in D1 VS D4, D4 VS D6 and D1 VS D6 comparisons were mapped to reference canonical pathways in KEGG database, and compared with whole transcriptome background (Additional file 9). The DEGs between D1 and D4 stages were assigned to 112 KEGG pathways. However, no significant gene enrichment was observed for any pathway (corrected *p*-value < 0.05). In the top five most enriched pathways, amino acid metabolism represented the dominant terms with most of gene down-regulated. Notably, “terpenoid backbone biosynthesis” with 30 members was also included in top five most enriched pathways. In the comparison of D4 and D6 stages, the pathways “proteasome”, “valine, leucine and isoleucine degradation” and “flavone and flavonol biosynthesis” were significantly enriched. These enrichments provide new insights into dynamic changes of specific biological process during petal development in *H. coronarium*.

### Analysis of putative genes related to terpene biosynthesis

**Fig. 4:**
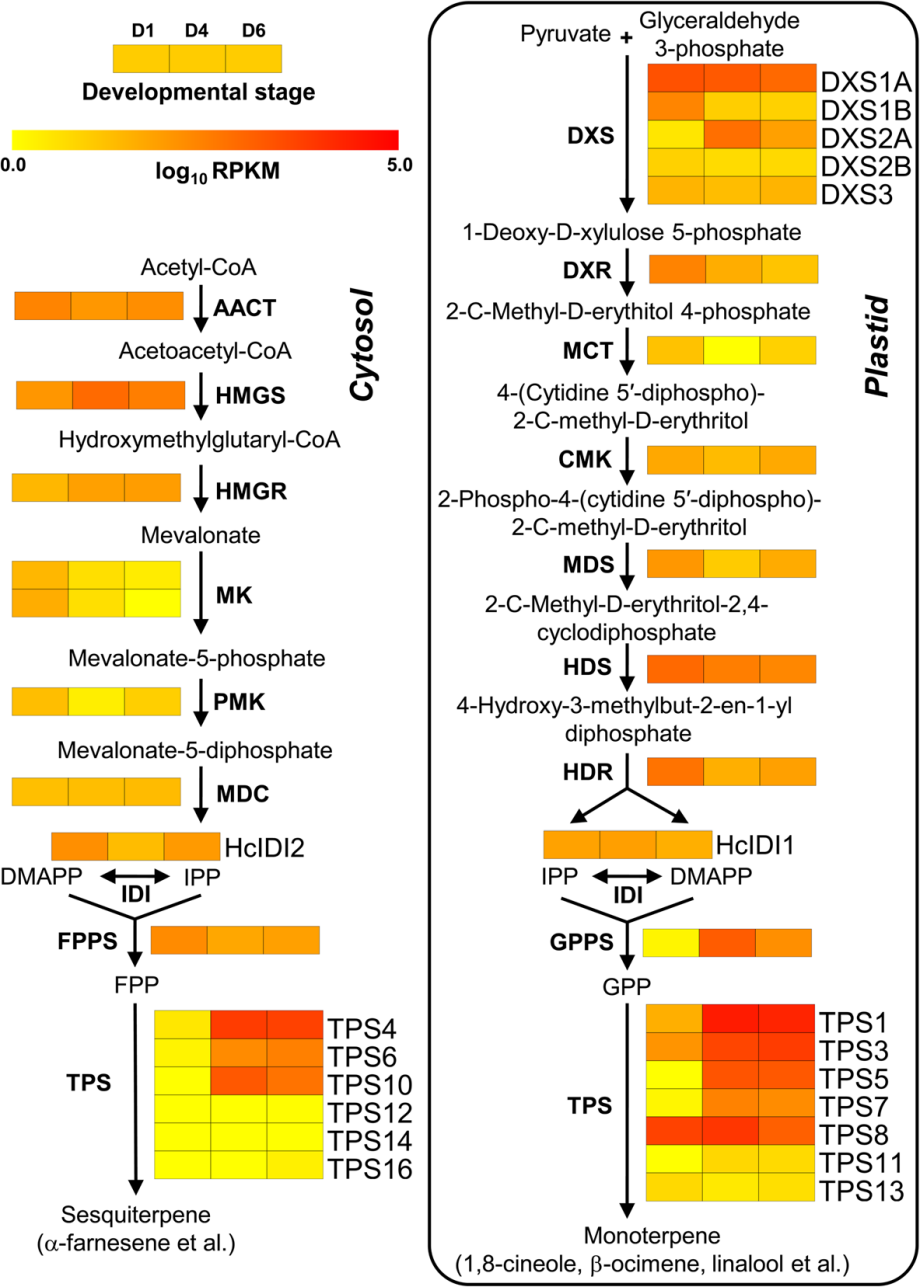
Expression patterns of genes encoding enzymes possibly involved in the monoterpenes and sesquiterpenes biosynthesis. The abbreviated name of enzyme in each catalytic step is showed in bold. Gene expression levels (log_10_ RPKM) in three petal developmental stages in *H. coronarium* (D1: squaring stage; D4: blooming stage; D6: senescence stage) are represented by color gradation. Gene expression with RPKM ≤ 1 was set to 0 after log_10_ transformation. Genes with more than one homology are represented by equal colored horizontal stripe and are termed from top to bottom in Arabic numerical order unless otherwise labeled. Possibly crosstalk between cytosol and plastid was not shown. HcIDI1 was predicted to target to plastids or mitochondria. The full names of enzymes are listed in the section of “Abbreviations”

**Fig. 5:**
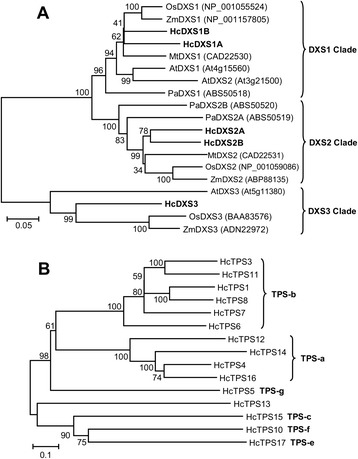
Phylogenetic analysis of DXSs and TPSs in *H. coronarium*. **a** Phylogenetic tree of plant DXSs based on the neighbor-joining method. Five DXSs (designated as HcDXS1-5) from *H. coronarium* are in bold. The scale bar indicates 5 % sequence divergence. GenBank accession numbers are shown in parentheses. At, *Arabidopsis thaliana*; Mt, *Medicago truncatula*; Os, *Oryza sativa*; Pa, *Picea abies*; Zm, *Zea mays*. **b** Phylogenetic tree of *H. coronarium* TPSs based on the neighbor-joining method. All the TPSs are classified into seven subfamilies (TPS-a through TPS-g). The scale bar indicates 10 % sequence divergence. The numbers at each branch indicate bootstrap percentages from 1000 replicates

To further detail the terpene biosynthesis in *H. coronarium* and identify the committed gene in the pathway, the putative genes related to terpene biosynthesis were mined according to the KEGG annotation and local TBLASTX search. Their expression levels were analyzed subsequently. In plants, monoterpenes are usually generated via the MEP pathway with seven enzymatic steps in plastid [52, 53]. Eleven complete genes encoding enzymes involved in the MEP pathway were identified in *H. coronarium* transcriptome, including five 1-deoxy-D-xylulose 5-phosphate synthase (DXS) genes, one each of 1-deoxy-D-xylulose 5-phosphate reductoisomerase (DXR) gene, 2-C-methyl-D-erythritol 4-phosphate cytidylyltransferase (MCT) gene, 4-(cytidine 5’-diphospho)-2-C-methyl-D-erythritol kinase (CMK) gene, 2-C-methyl-D-erythritol-2,4-cyclodiphosphate synthase (MDS) gene, 4-hydroxy-3-methylbut-2-en-1-yl diphosphate synthase (HDS) gene, 4-hydroxy-3-methylbut-2-en-1-yl diphosphate reductase (HDR) gene (Fig. 4). All enzymes encoded by these eleven genes were predicted to target to plastids (Fig. 4), consistent with the localization of MEP pathway enzymes in Arabidopsis [54]. DXS catalyses a rate-limiting step of the MEP pathway, which converts the pyruvate and glyceraldehyde 3-phosphate into 1-deoxy-D-xylulose 5-phosphate [55, 56]. Phylogenetic analysis of plant DXSs showed that HcDXS1A and HcDXS1B belonged to DXS1 clade (Fig. 5a), proteins in which possibly have the housekeeping function; HcDXS2A and HcDXS2B clustered into DXS2 clade, which was proposed to be involved in secondary isoprenoid metabolism; HcDXS3 was a member of DXS3 clade, which might be related to the synthesis of some products essential for plant survival and required at lower levels such as gibberellic acid and abscisic acid [57–59]. Expression analysis of HcDXSs by RNA-Seq revealed that the expression profile of *HcDXS2A* exhibited a similar pattern with the emissions of some monoterpenes (Figs. 1 and 4). The transcripts of *HcDXS1A, HcDXS2B* and *HcDXS3* stayed at a relative steady level during petal development (Fig. 4). However, the expression levels of *HcDXS1B* and the following six genes in the MEP pathway were significantly down-regulated from squaring stage (D1) to blooming stage (D4) and still stayed at medium or high levels (Fig. 4), possibly reflecting the declining demand for metabolic flux toward primary metabolism.

In plants, the MVA pathway, mainly located in cytosol, provides precursors for cytosolic and mitochondrial isoprenoids including sesquiterpenes, sterols and ubiquinones [8]. In *H. coronarium* transcriptome, seven complete genes related to the MVA pathway were identified including acetyl-CoA acetyltransferase (AACT) gene, hydroxymethylglutaryl-CoA synthase (HMGS) gene, hydroxymethylglutaryl-CoA reductase (HMGR) gene, phosphomevalonate kinase (PMK) gene, mevalonate diphosphate decarboxylase (MDC) gene and two mevalonate kinase (MK) genes (Fig. 4). The expression of *HcHMGS* and *HcHMGR* increased 7.42 and 2.81-fold from D1 to D4 stage, respectively, thereafter the expression of *HcHMGS* declined by 59.08 %, while *HcHMGS* increased 1.14-fold (Fig. 4). Different from *HcHMGS* and *HcHMGR*, the expression levels of *HcAACT*, *HcMK1/2* and *H*cPMK were significantly down-regulated from D1 to D4 stage.

Isopentenyl diphosphate isomerase (IDI) converts IPP to DMAPP in a reversible reaction and regulates the equilibrium between IPP and DMAPP [8]. Two *IDI* genes were found in *H. coronarium* transcriptome (Fig. 4). Protein subcellular localization prediction revealed HcIDI2 were localized in cytosol, while HcIDI1 in plastids or mitochondria (Fig. 4). The transcript level of *HcIDI1* remained at high level and did not show significant changes in three stages. However, the expression quantity of *HcIDI2* decreased 87.08 % from D1 to D4 stage and rose 4.66-fold from D4 to D6 stage. After the formation of IPP and DMAPP, short-chain prenyltransferases (isoprenyl diphosphate synthases) consisted of GPP synthase (GPPS), FPP synthase (FPPS) and GGPP synthase (GGPPS) catalyze the head-to-tail condensation of IPP and DMAPP to produce prenyl diphosphate GPP, FPP and GGPP, the precursors of mono-, sesqui- and diterpenes, respectively [60]. Only one FPPS exist in *H. coronarium* transcriptome and has been demonstrated to involve in floral volatile sesquiterpene biosynthesis [45]. Also, one GPPS (HcGPPS) was identified from the current assembly transcriptome. Expression analysis revealed that the expression profile of *HcGPPS* has a similar trend with the emission of monoterpenes and show noticeable petal developmental control (Fig. 4).

**Fig. 6:**
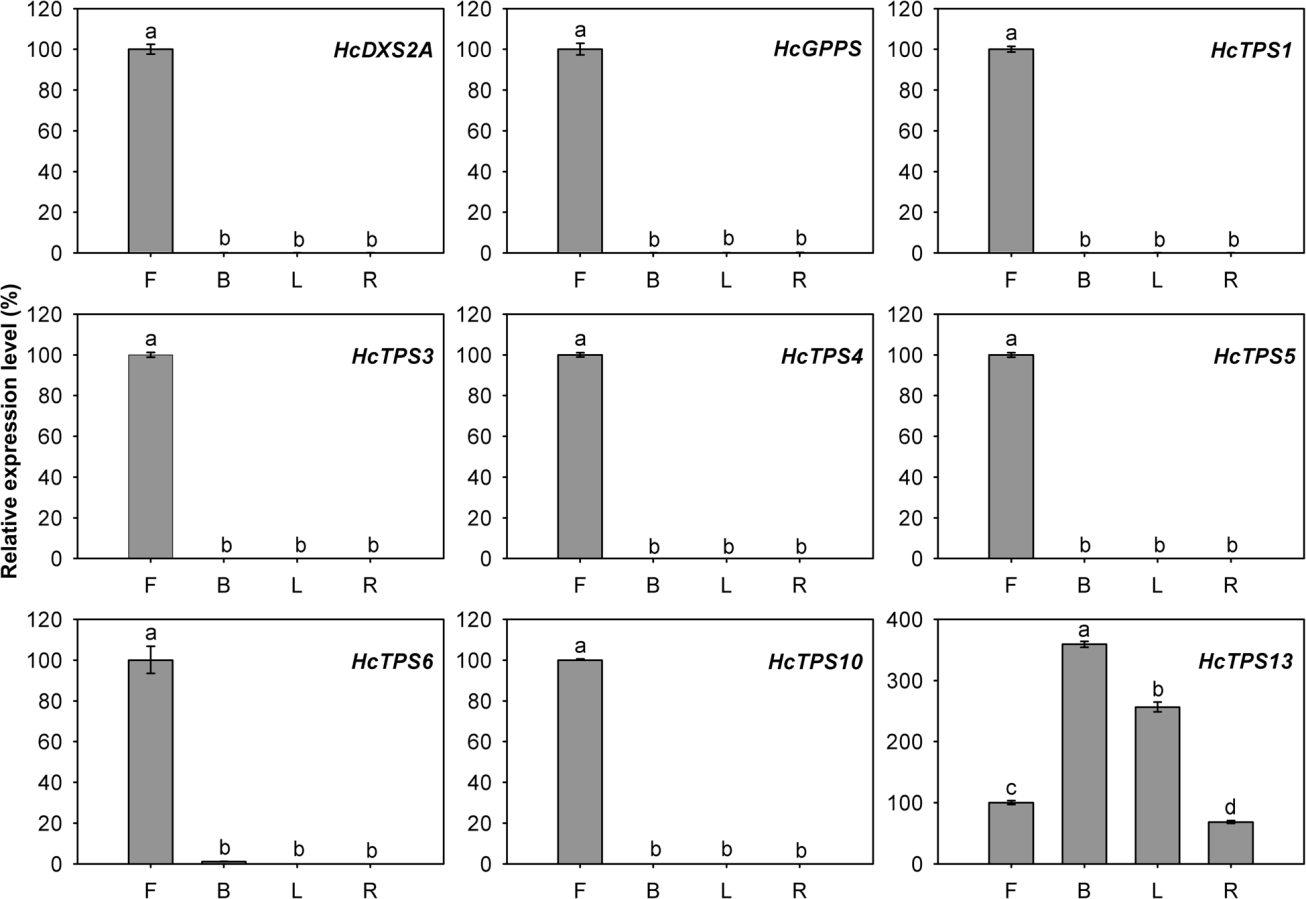
Expression analysis of terpene biosynthetic genes in different tissues. Relative transcription level of flowers was set to 1 (100 %). Error bars indicate the calculated maximum and minimum expression quantity of three replicates. Different lowercase letters labeled on bars indicate statistically significant differences at the level of *P* < 0.05. F, flowers; B, bracts; L, leaves; R, rhizomes

Following the formation of the prenyl diphosphate precursors, an array of structurally diverse cyclic and acyclic monoterpenes and sesquiterpenes are generated through the action of TPSs, which directly determine product specificity [9]. There are 15 complete and two partial TPSs were identified through the sequence homology-based search in the current assembled *H. coronarium* transcriptome. Among them, HcTPS7 and HcTPS8 which involved in floral scent formation were characterized as sabinene and linalool synthase [46]. Based on amino acid sequence relatedness, plant TPSs are classified into seven different subfamilies, designated TPS-a through TPS-g [15, 61]. Phylogenetic analysis revealed that HcTPS1, HcTPS3, HcTPS6-8 and HcTPS11 belonged to TPS-b subfamily, which predominantly consists of angiosperm mono-TPSs; HcTPS4, HcTPS12, HcTPS14 and HcTPS16 clustered into TPS-a subfamily, which is mainly composed of angiosperm sesqui-TPSs; HcTPS5 was a member of TPS-g subfamily, which produce acyclic terpenes; HcTPS10, HcTPS15 and HcTPS17 belonged to TPS-f, TPS-c and TPS-e subfamily, respectively (Fig. 5b). HcTPS15 and HcTPS17 were the orthologs of copalyl synthase and kaurene synthase respectively, which involved in biosynthesis of gibberellins. Interestingly, HcTPS13 did not fall into any of the six previously defined angiosperm TPS subfamilies, but was closely related to the gymnosperm-specific subfamily TPS-d (Fig. 5b and Additional file 10). The full-length cDNA of HcTPS13 contained putative ORF of 1,833 bp encoding 611 amino acid residues. The motif DDXXD, which is highly conserved in almost all TPSs [62], is also present in HcTPS13 (Additional file 11). Alignment of the amino acid sequence showed that HcTPS13 share very low identity (<23 %) with other TPSs in *H. coronarium*. Then, the deduced amino acid sequence of HcTPS13 was used to BLASTP search in NCBI Nr database and four *Phoenix dactylifera* TPSs were matched. They show 40 %-44 % sequence identity and 58 %-64 % sequence similarity to HcTPS13 (Additional file 12). Phylogenetic analysis revealed that HcTPS13 and four *P. dactylifera* TPSs were distinctly clustered in a novel monophyletic branch (Additional file 10). Protein subcellular localization prediction showed that HcTPS4, HcTPS6, HcTPS10, HcTPS12, HcTPS14 and HcTPS16 were localized in the cytosol, while HcTPS1, HcTPS3, HcTPS5, HcTPS11 and HcTPS13 were targeted to the plastid (Fig. 4). Expression analysis of HcTPSs by RNA-Seq revealed that expression of HcTPS4, HcTPS6 and HcTPS10 displayed positive correlation with the emission of sesquiterpene α-farnesene, while HcTPS1, HcTPS3 and HcTPS5 have similar trend with the emission of monoterpenes (Figs. 1 and 4). However, only low expression levels for HcTPS10-14 and HcTPS16 were measured (Fig. 4), suggesting other potential functions rather than the synthesis of floral volatile terpenes. Q-PCR analysis showed that all key genes mined above were expressed specifically in flowers and almost undetectable in bracts, leaves and rhizomes (Fig. 6), indicating their close correlation with the emission of terpenes. However, *HcTPS13* gene were expressed constitutively in *H. coronarium* and expressed highest in bracts (Fig. 6).

### Functional characterization of HcTPS6

**Fig. 7:**
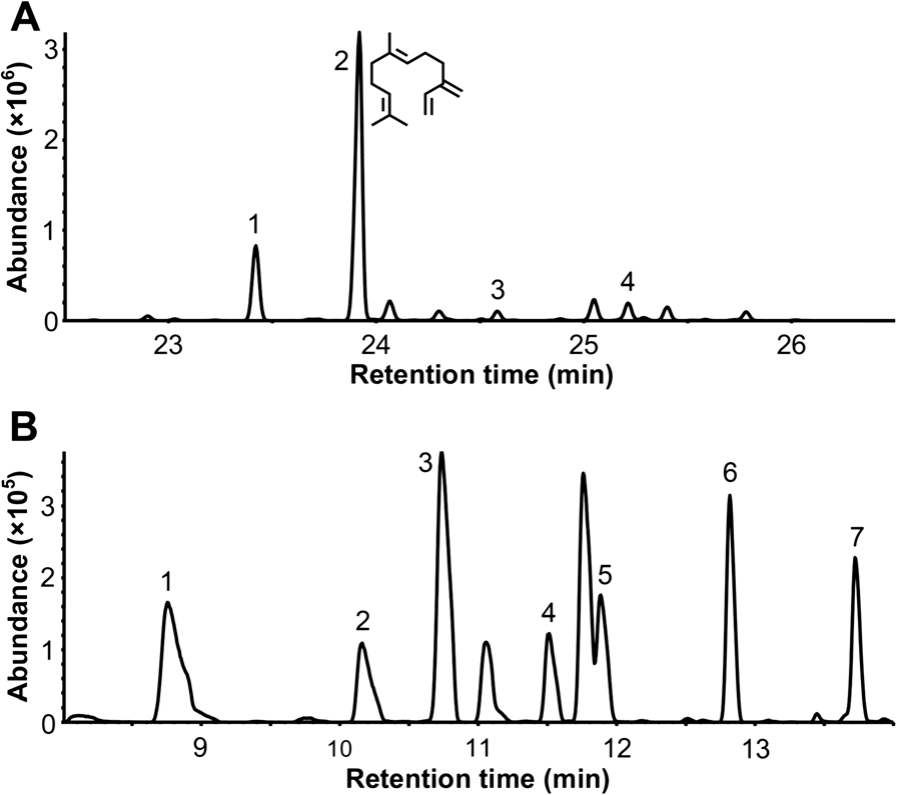
Analysis of products generated by HcTPS6 enzyme from FPP and GPP. **a** Total ion chromatogram of the sesquiterpene products formed by HcTPS6 from FPP. 1, α-bergamotene; 2, β-farnesene; 3, α-curcumene; 4, β-bisabolene. Chemical structure of β-farnesene was labeled on peak 2. Unlabeled peaks were unidentified sesquiterpene products. Mass spectra of peaks and standards are provided in Additional file 12. **b** Total ion chromatogram of the monoterpene products formed by HcTPS6 from GPP. 1, α-thujene; 2, sabinene; 3, myrcene; 4, α-terpinene; 5, limonene; 6, γ-terpinene; 7, terpinolene. Unlabeled peaks were contamination from PDMS fiber. Mass spectra of peaks and standards are provided in Additional file 12

Given that HcTPS6 belong to TPS-b subfamily (almost mono-TPSs), but it do not possess the plastidic signal peptide. Therefore, the catalytic function of HcTPS6 with FPP or GPP needs to be elucidated experimentally. The coding region of HcTPS6 was expressed in *Escherichia coli*, and the activity of purified recombinant protein was analyzed *in vitro*. When incubated with FPP, HcTPS6 synthesized β-farnesene (61.6 %) as its major product and 16 by-products, such as α-bergamotene (15.7 %), β-bisabolene (4.1 %) and α-curcumene (2.2 %) (Fig. 7). Therefore, HcTPS6 was designated as β-farnesene synthase. With GPP as substrate, HcTPS6 catalyzed the formation of α-thujene (20.1 %), sabinene (9.1 %), myrcene (26.7 %), α-terpinene (7.5 %), limonene (10.6 %), γ-terpinene (14.8 %) and terpinolene (11.2 %) (Fig. 7). All of these products were not found in compounds formed by the extracts of *E. coli* expressing empty vector in the presence of FPP/GPP (data not shown).

### Analysis of putative genes related to benzenoid biosynthesis

**Fig. 8:**
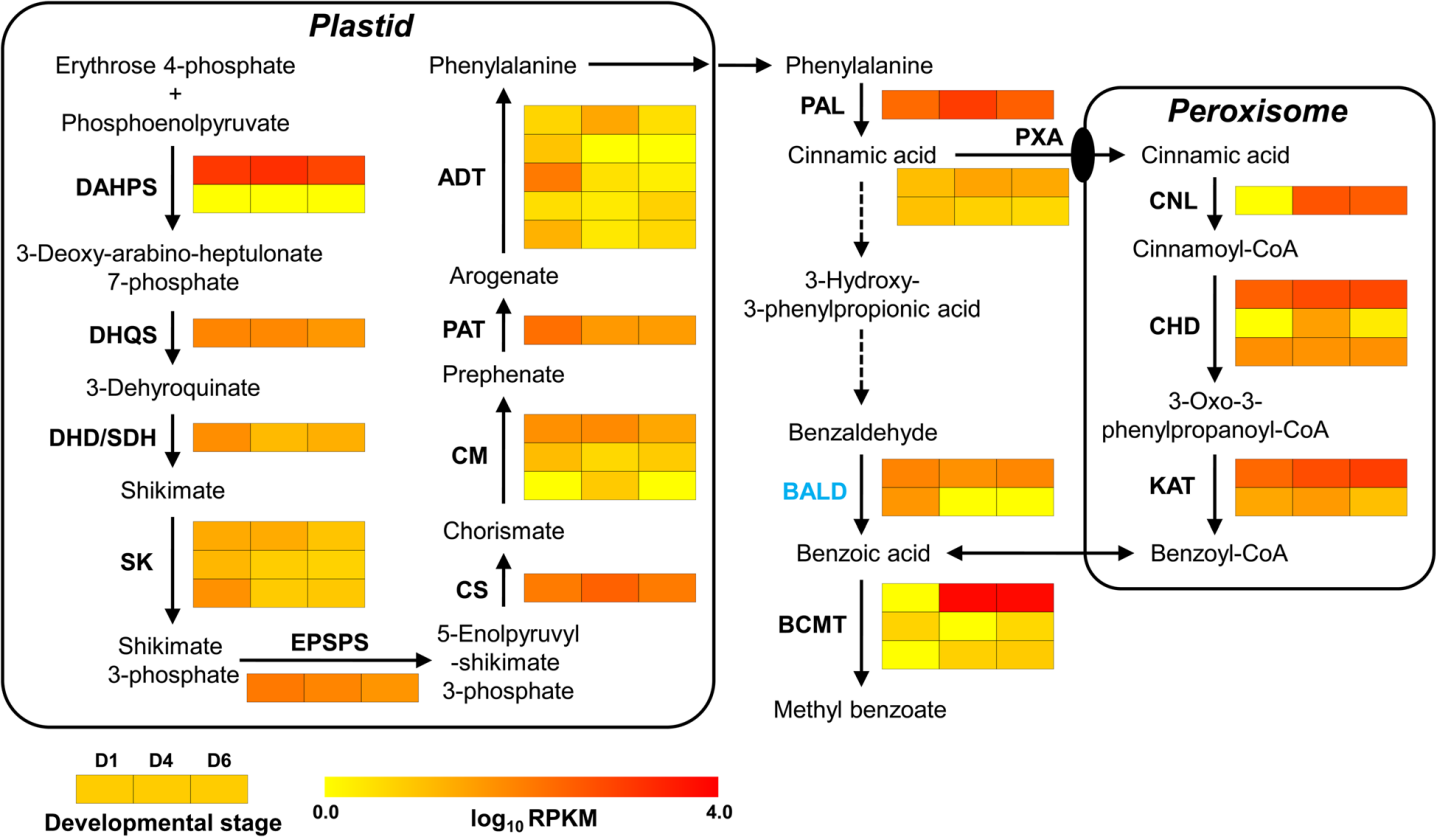
Expression patterns of genes encoding enzymes possibly involved in the shikimate/benzenoid pathway in *H. coronarium*. The abbreviated name of enzyme in each catalytic step is showed in bold. Gene expression levels (log_10_ RPKM) in three developmental stages (D1: squaring stage; D4: blooming stage; D6: senescence stage) are represented by color gradation. Gene expression with RPKM ≤ 1 was set to 0 after log_10_ transformation. The subcellular localization of BALD in blue was predicted as mitochondria. Broken arrows represent hypothetical steps not yet described in plant. Genes with more than one homology are represented by equal colored horizontal stripe and are termed from top to bottom in Arabic numerical order. The full names of enzymes are listed in the section of “Abbreviations”

Given that the shikimate and arogenate pathways provide the carbon flux for the biosynthesis of benzenoids in plant, the genes related to shikimate and arogenate pathways were first investigated. The shikimate pathway consists of seven reactions catalyzed by six enzymes in plant and originates from phosphoenolpyruvate (PEP) and erythrose 4-phosphate (E4P) to produce chorismate [63]. Nine complete shikimate pathway genes were identified in the transcriptome, including two 3-deoxy-7-phosphoheptulonate synthase (DAHPS) genes, three shikimate kinase (SK) genes and one each of 3-dehydroquinate synthase (DHQS) gene, dehydroquinate dehydratase/shikimate dehydrogenase (DHD/SHD), 3-phospho shikimate 1-carboxyvinyltransferase (EPSPS) gene and chorismate synthase (CS) gene (Fig. 8). The expression levels of these genes at three stages were showed in Fig. 8. Among them, DAHPS is the first committed enzyme in shikimate pathway and controls the overall carbon flux into the pathway [64]. The *HcDAHPS1* gene displayed a very high expression level during the petal development, while the expression level of *HcDAHPS2* was low, suggesting that *HcDAHPS1* play a key role in regulating the carbon flux into the shikimate pathway. Following the formation of chorismate, phenylalanine was synthesized via the arogenate pathway, including three chorismate mutases (CMs), one prephenate aminotransferase (PAT) and five arogenate dehydratases (ADTs) in *H. coronarium*. In five *ADT* genes, only *HcADT1* was up-regulated significantly in the process of D1 to D4, while *HcADT2-4* was down-regulated. All aforementioned enzymes were predicted to be plastidial localization (Fig. 8).

**Fig. 9:**
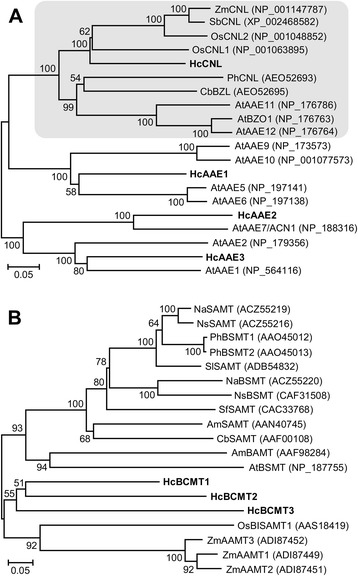
Phylogenetic analysis of AAEs and BCMTs in *H. coronarium*. **a** Phylogenetic tree of clade VI AAEs based on the neighbor-joining method. CNL orthologs are shadowed in grey. **b** Phylogenetic tree of HcBCMTs with other functional characterized plant benzenoid carboxyl methyltransferases (BCMTs). Proteins identified from *H. coronarium* are in bold. The scale bar indicates 5 % sequence divergence. GenBank accession numbers are shown in parentheses. The numbers at each branch indicate bootstrap percentages from 1000 replicates. At, *Arabidopsis thaliana*; Am, *Antirrhinum majus*; Cb, *Clarkia breweri*; Na, *Nicotiana alata*; Ns, *Nicotiana suaveolens*; Os, *Oryza sativa*; Ph, *Petunia hybrid*; Sb, *Sorghum bicolor*; Sf, *Stephanotis floribunda*; Sl, *Solanum lycopersium*; Zm, *Zea mays*

To further analyze the process of benzenoid biosynthesis in *H. coronarium*, and to identify the committed genes in the pathway, the putative genes encoding enzymes involved in the benzenoid biosynthesis were sought using the local TBLASTX search. The first committed step in benzenoid biosynthesis is catalyzed by PAL, which deaminates phenylalanine to cinnamic acid [21]. One *PAL* gene existed in the current assembled transcriptome and its expression was significantly regulated by petal development (Fig. 8). Then, the formation of benzenoids from cinnamic acid proceeds via β-oxidative pathway and non β-oxidative pathway [22]. The β-oxidative pathway in petunia flowers needs four reactions catalyzed by three enzymes, including Cinnamate:CoA ligase/acyl-activating enzyme (CNL/AAE), cinnamoyl-CoA hydratase-dehydrogenase (CHD) and 3-ketoacyl CoA thiolase (KAT) [23–26]. Using characterized petunia genes to BLASTX search the *H. coronarium* transcriptome, four *CNL/AAEs*, three *CHDs* and two *KATs* were found. The superfamily AAE comprises carboxyl-CoA ligases and related protein in plant, and contains seven phylogenetic clades [65]. Phylogenetic analysis of the clade VI AAEs revealed that only one out of four *H. coronarium* AAEs, HcCNL, fell into the group of PhCNL orthologs [23] and was closely related to CNLs in other monocots (Fig. 9a). The expression pattern of *HcCNL* showed positive correlation with the emission of methyl benzoate (Fig. 1g and Fig. 8). For *HcCHDs*, the expression levels of *HcCHD1* and *HcCHD2* were significantly up-regulated from D1 to D4 stage, whereas *HcCHD3* is constitutively expressed at three stages (Fig. 8). The expression of *HcKAT1* escalated slightly from D1 to D6 stage, while *HcKAT2* showed insignificant change (Fig. 8). Besides, two peroxisomal ATP-binding cassette transporters (PXAs), homologs of Arabidopsis CTS/PXA1 [27], were identified and might account for the transport of cinnamic acid into peroxisome in *H. coronarium*. To non-β-oxidative pathway, NAD-dependent benzaldehyde dehydrogenase (BALD) accounted for the oxidation of benzaldehyde into benzoic acid [28]. Two *BALD* homologs in *H. coronarium* were searched using the characterized snapdragon *BALD* gene as query sequence [28]. Different from snapdragon *BALD*, *HcBALDs* did not show correlation with the emission of benzenoids (Fig. 8).

In the final step, methyl benzoate is synthesized from benzoic acid and *S*-adenosyl-L-methionine (SAM), and catalyzed by benzenoid carboxyl methyltransferase (BCMT) including benzoic acid carboxyl methyltransferase (BAMT) and benzoic acid/salicylic acid carboxyl methyltransferases (BSMT) [66, 67]. Phylogenetic analysis of three identified HcBCMTs with other plant BCMTs showed that three identified HcBCMTs are closely related to monocot rice salicylic acid methyltransferase (SAMT) and maize anthranilic acid methyltransferase (AAMT). It also showed that those identified HcBCMTs are distant from other characterized BAMTs and BSMTs in dicots (Fig. 9b). The expression pattern of *HcBCMT1* was closely correlated with the emission of methyl benzoate, while the expression of *HcBCMT2/3* remained at low levels (Fig. 1g and Fig. 8).

**Fig. 10:**
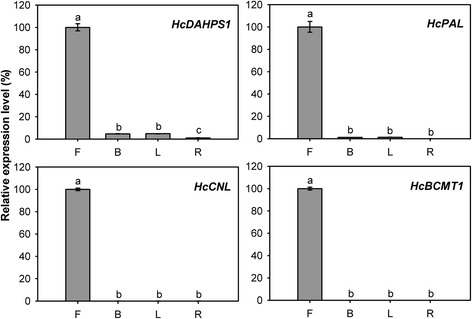
Expression analysis of benzenoid biosynthetic genes in different tissues. Relative transcription level of flowers was set to 1 (100 %). Error bars indicate the calculated maximum and minimum expression quantity of three replicates. Different lowercase letters labeled on bars indicate statistically significant differences at the level of *P* < 0.05. F, flowers; B, bracts; L, leaves; R, rhizomes

Q-PCR analysis showed that *HcDAHPS1* and *HcPAL* were expressed preferentially in flowers and expressed at lower levels in bracts, leaves and rhizomes. Meanwhile, *HcCNL* and *HcBCMT1* were specifically expressed in flowers and almost undetectable in bracts, leaves and rhizomes (Fig. 10). The genes expression was consistent with the flower-specific release of methyl benzoate.

### Analysis of TFs

**Fig. 11:**
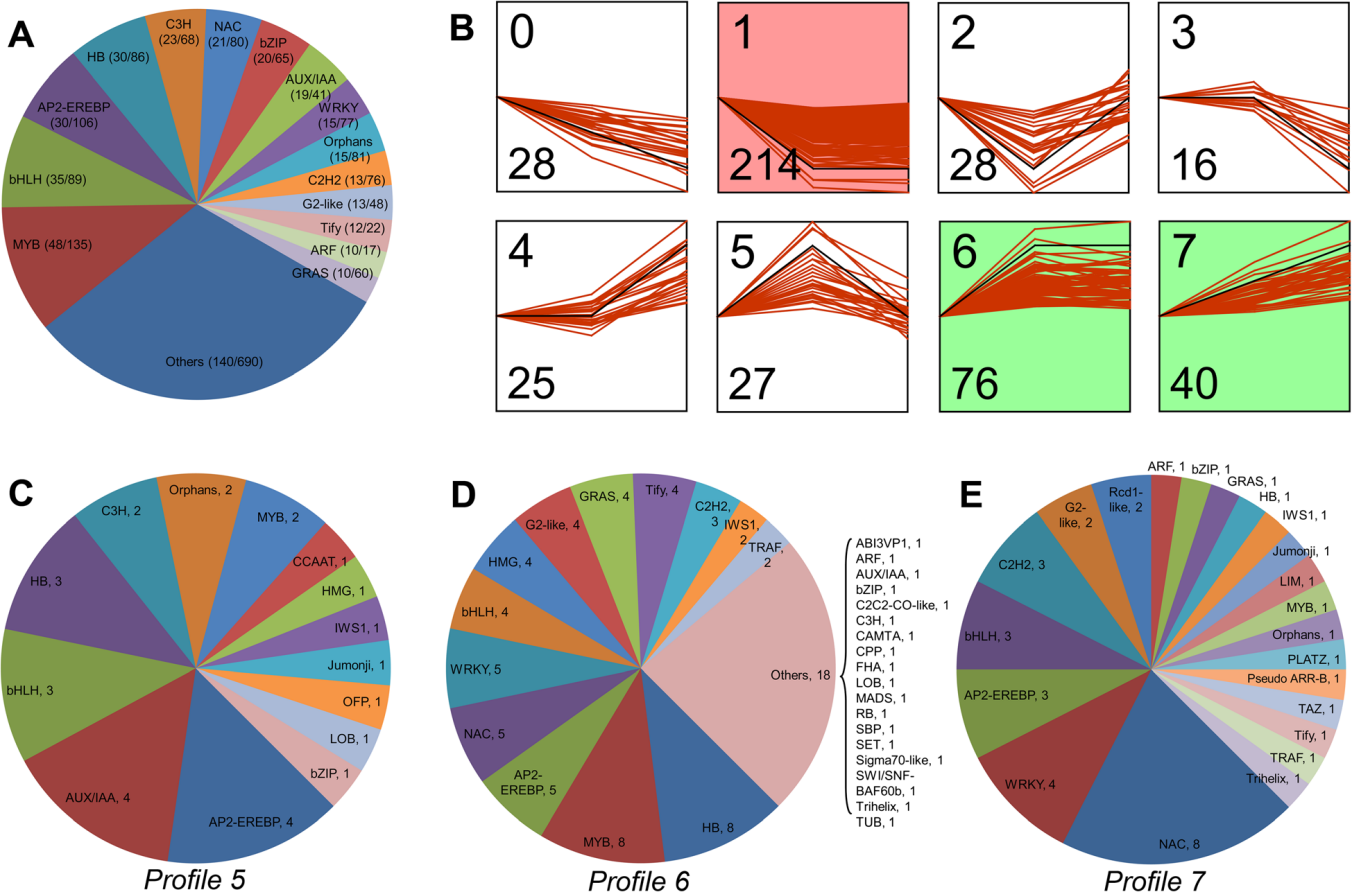
Analyses of differentially expressed TFs during petal development. **a** Classification of differentially expressed TFs. The differentially expressed TFs are obtained from D1 VS D4 or D4 VS D6, or both. The numbers in the parentheses after each TF family indicate the number of changed TFs in this family (first number) and the total number of this TF family identified by RNA-Seq (second number). **b** Cluster analysis of differentially expressed TFs. All differentially expressed TFs were divided into 8 distinct temporal expression profiles using STEM software. Profiles in color indicate statistically significant (*P* < 0.01) (Green, up-regulated; red, down-regulated). The number in the top left hand corner is the profile ID number. Number of assigned genes of the profile is shown in the lower left hand corner. The black lines show model expression profiles. The red lines represent individual gene expression profiles. The x-axis represents the developmental stages (D1, D4 and D6). The time series were transformed to start at 0 with log_2_(RPKM + 1) normalization. **c**, **d** and **e** Classification of TFs assigned in the profile 5, 6 and 7. Number after each TF family indicates the TF number of this family

TFs play a major role in regulating the genes expression of various plant developmental and physiological processes, including plant secondary metabolism [68, 69]. In this study, we identified 1,741 TFs (including transcriptional regulators), representing 2.65 % of *H. coronarium* unigenes and falling into 80 TF families classified by PlnTFDB database [70] (Fig. 11a). The most abundant TF family is MYB (135), followed by AP2-EREBP (106), bHLH (89), HB (86), Orphans (81), NAC (80), WRKY (77), C2H2 (76), C3H (68) and bZIP (65) (Fig. 11a). Among them, there are 454 TFs showed differential expression in D1 VS D4 or D4 VS D6, or both (Fig. 11a). These TFs were classified into 64 TF families and were likely to regulate the expression changes of structural genes related to various biological processes during the petal development. The MYB family (48) represented the largest amount of differentially expressed TFs, followed by bHLH (35), AP2-EREBP (30), HB (30), C3H (23), NAC (21), bZIP (20), AUX/IAA (19) and WRKY (15).

STEM cluster analysis of differentially expressed TFs revealed eight expression profiles, including three significant expression profiles (profile 1, 6 and 7) (Fig. 11b). Since the expression patterns of profile 5, 6 and 7 showed positive correlation with the emission of volatile compounds, the TFs in these profiles were further analyzed. In profiles 5 (0, 1, 0), there are 27 TFs distributed in 14 TF families, including AP2-EREBP (4), AUX/IAA (4), bHLH (3), HB (3), C3H (2), Orphans (2), MYB (2), etc. (Fig. 11c). 76 TFs clustered in profiles 6 (0, 1, 1) and classified into 32 TF families (Fig. 11d). Families MYB and HB with eight members represented the most abundant TF families, followed by AP2-EREBP (5), NAC (5), WRKY (5) and bHLH (4). There are 40 TFs clustered in profiles 7 (0, 1, 2) and grouped into 22 TF families, covered eight NACs, four WRKYs, three AP2-EREBPs, three bHLHs, three C2H2s, etc. (Fig. 11e).

**Fig. 12:**
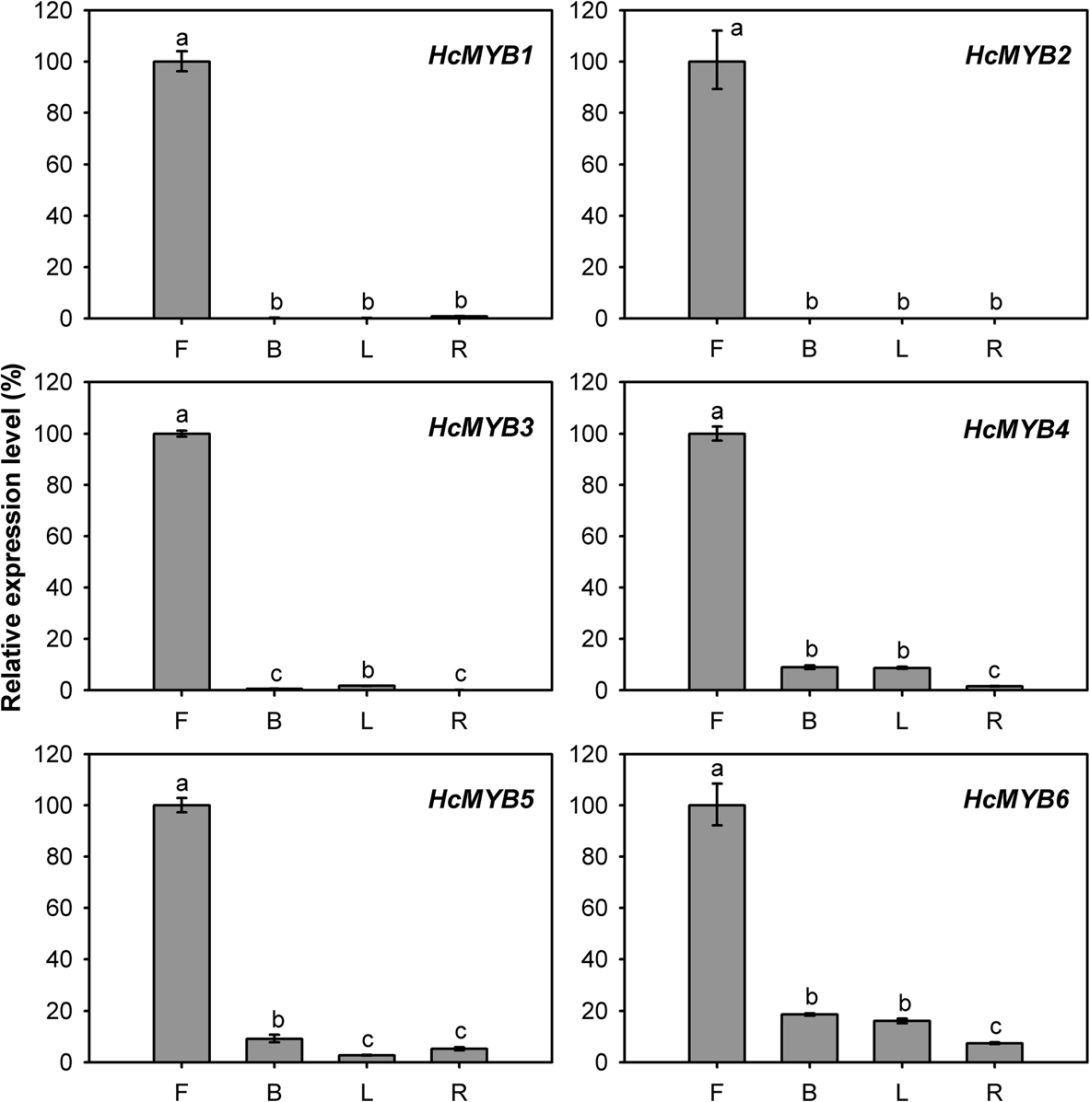
Expression analysis of *HcMYB1-6* in different tissues. Relative transcription level of flowers was set to 1 (100 %). Error bars indicate the calculated maximum and minimum expression quantity of three replicates. Different lowercase letters labeled on bars indicate statistically significant differences at the level of *P* < 0.05. F, flowers; B, bracts; L, leaves; R, rhizomes

We first focused on MYB TF family, in which the scent related TFs were identified in other plants [29, 30, 32]. Out of 13 MYBs clustered in profile 5, 6, and 7, six were R2R3-MYBs, which were reported to involve in the control of secondary metabolism [71]. To understand the spatial expression profiles, Q-PCR analysis of six R2R3-MYBs (designated as *HcMYB1-6*) were performed in different organs. The results showed that expression of *HcMYB1* and *HcMYB2* were flower specific. No or only negligible levels of their transcripts were detected in bracts, leaves and rhizomes (Fig. 12). The flower-specific and developmental expression pattern is one feature of known scent-related R2R3-MYB TFs [29, 30, 32]. For *HcMYB3-6*, the expression quantities were highest in flowers, while the expression levels were low in bracts, leaves and rhizomes (Fig. 12).

### Gene expression validation

**Fig. 13:**
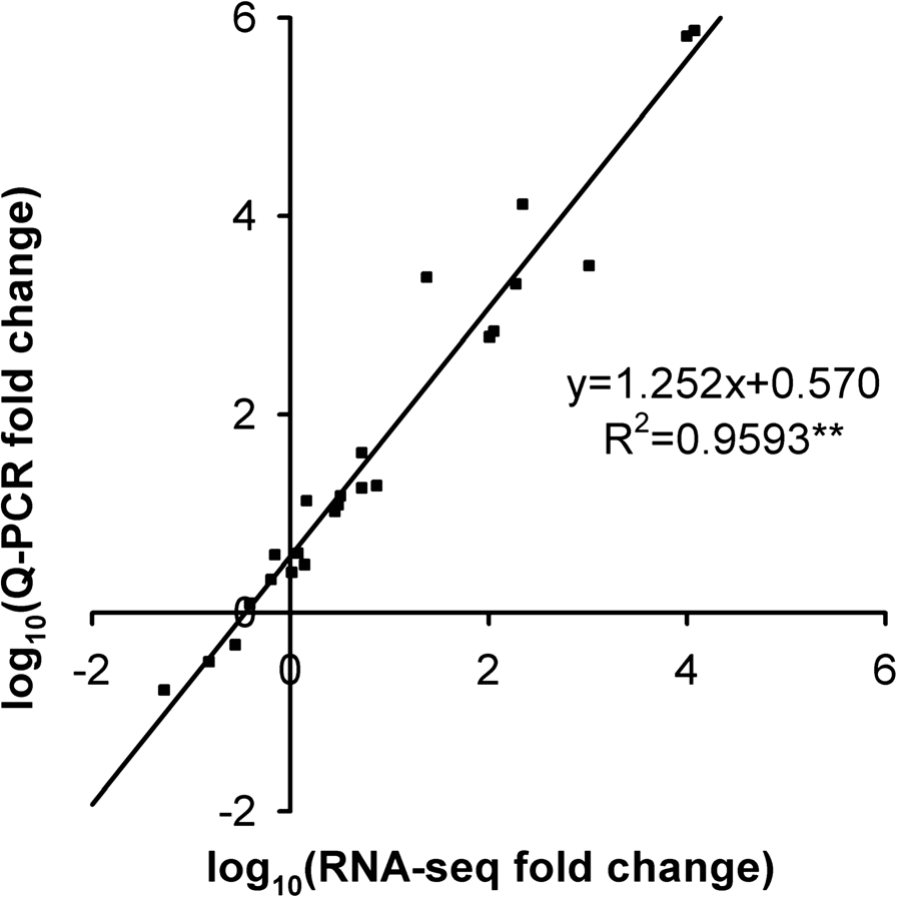
Correlation analysis of fold change values obtained from RNA-Seq and Q-PCR. RNA-Seq fold change refers to the ratios of RPKM values of D4 (D6) to D1 for selected transcripts, while Q-PCR fold change is the relative quantity of D4 (D6) normalized to expression level of D1. ^**^ indicates a significant correlation at *P* < 0.01

To experimentally validate the genes expression levels during petal development, twelve unigenes related to terpene and shikimate/benzenoid biosyntheses were selected for real-time quantitative PCR (Q-PCR) analysis. The results showed that the genes expression profiles obtained by Q-PCR were largely consistent with those measured by DGE profiling (Additional file 13). Linear regression analysis revealed the fold change values of Q-PCR and RNA-Seq showed a strong positive correlation (*R*^*2*^ = 0.9593) at the level of *P* ≤ 0.01 (Fig. 13). These results demonstrate the credibility of RNA-Seq data generated in this study.

## Discussion

Nowadays, next-generation sequencing technology is widely applied to transcriptome profiling investigation in the non-model plant, providing valuable resources for functional genomics research. In Zingiberaceae family, based on RNA-Seq technology, leaves transcriptome of *Costus pictus* with 55,006 transcripts [72] and rhizomes transcriptomes of three varieties of *Curcuma longa* with 56,770-91,958 transcripts [73] were reported, providing insights into the synthesis of pharmacologically active metabolites. Moreover, sequencing the leaves transcriptomes of wilt-sensitive ginger and wilt-resistant mango ginger using Illumina sequencing technology led to 36,359 and 32,312 assembled transcript sequences, respectively [74]. Comparative analysis of two transcriptomes identified several candidate genes for resistance to bacterial wilt pathogen in mango ginger. In this study, using Illumina platform, more than 81 M high-quality reads were generated and were further assembled into 65,591 *H. coronarium* unigenes. Meanwhile, 50.90 % of the total unigenes were annotated by public databases. The Q20 of sequencing is 97.67 % and the N50 of assembled transcriptome is 1,284 bp, mirroring high-quality of the transcriptome (Table 1). Also, many complete genes have been isolated based on the information of the transcriptome and verified by Sanger sequencing (data no shown), indicating the intact assembly. To the best of our knowledge, this is the first report of large scale transcriptome data in the genus of *Hedychium*. The data provides an important resource for insight into the specific biological process and facilitates gene discovery and their functional studies in *Hedychium* species. In addition, the medicinal properties of *H. coronarium* leaves and rhizomes are determined by some secondary metabolites. This transcriptome, which covers the sequence information of leaves and rhizomes, also lays the basis for investigating the biosynthesis of these medicinal components.

Floral scent is determined by a complex mixture of low-molecular-mass volatile molecules, primarily consisting of terpenoids and benzenoids/phenylpropanoids. Combination of metabolic and gene expression profiling have proven extremely powerful to identify candidate genes and enzymes involved in the formation of compounds that contribute to floral fragrance. In Arabidopsis, Chen and colleagues first detected the emission profiles of terpenes from Arabidopsis flowers. And then, upon the genomic information and RT-PCR method, the expression profile analysis of all *AtTPS* genes revealed several completely or almost completely flower-specific candidate *AtTPS* genes [12]. When no complete genome sequence was available, expressed sequence tag (EST) databases were used as the source of genomic sequence information in previous studies. For example, the petals of two rose cultivars with very distinct volatile profiles were applied to construct an EST database with 2,873 individual clones. Then, the expression profiles of 350 selected unique genes in two cultivars and two petal developmental stages were compared using DNA microarrays, resulting in the discovery of several novel floral fragrance-related genes [14]. This approach of EST database combined with metabolic profiling and DNA microarrays were also used in petunia and snapdragon to identify novel scent-related candidate structural genes or TFs [29, 75, 76]. In this study, using the next-generation technology, the formation of floral scent in a monocot was first investigated comprehensively at the transcriptome level. We identified 35 and 33 candidate genes covered all known enzymatic steps in the biosynthesis of volatile terpenes and benzenoids, respectively (Figs. 4 and 8). These candidate genes and their expressions provide a global overview of the formation of floral fragrance in *H. coronarium* (Figs. 4 and 8). Combining with the chemical analysis of volatile composition (Fig. 1), several candidate genes whose expression coincided with scent production, might play critical roles in regulating the formation of floral scent.

In plants, the regulation of isoprenoid pathway is complex, while the fluxes are controlled at the transcript level to a large extent [8]. It has been demonstrated that DXS, the first enzyme of MEP pathway, plays an important role in the overall regulation of metabolic flux in this pathway [56]. Thus, understanding the function of this enzyme is valuable for the potential modulation of the terpene production. In *H. coronarium*, five *HcDXSs* displayed differential expression patterns, suggesting their distinct functions *in vivo*. When the transcript level of *HcDXS1A/B* declined, *HcDXS2A*, a member of DXS2 clade gene, increased sharply from D1 to D4 and was regulated developmentally (Fig. 4). It is likely that the rise of *HcDXS2A* transcript guarantee sufficient metabolic flux toward volatile terpenes during petal development. In Norway spruce, DXS1 clade gene is constitutively expressed, while DXS2 clade genes show increased transcript abundance after treatments, suggesting distinct functions of the DXS genes in primary and secondary terpenoid metabolism [58]. GPPS provides the branch-point intermediate GPP for terpene biosynthesis, and controls the carbon flux into various monoterpene products. In *Phalaenopsis bellina*, a flower-specific homodimeric PhGPPS, displaying a closely related expression pattern with the emission of monoterpenes, suggested that it plays a crucial role in the regulation of volatile monoterpenes productions in orchid flowers [77]. Likewise, the gene expression of *HcGPPS* coincided with some monoterpenes production, indicating its regulatory role in floral volatile monoterpene biosynthesis during the petal development.

TPSs are highly diversified throughout the plant kingdom and form a mid-size gene family, which are directly responsible for the synthesis of the various terpenes [78]. The genomes of some model angiosperms possess 24–69 full length TPS genes [78], while *H. coronarium* transcriptome contains 15 complete and two partial TPSs. There could be more TPS genes in the genome of *H. coronarium*. Because some TPS genes display tissue-specific and inducible expression patterns [61], leading to the lack in current transcriptome. The fifteen complete TPSs distribute to all known angiosperm clades (Fig. 5b). Intriguingly, HcTPS13 and four monocot *P. dactylifera* TPSs with DDXXD motif (class I type TPS) form a novel monophyletic branch that is close to the gymnosperm-specific subfamily TPS-d (Additional file 10), reflecting the evolutionary diversity and complexity of TPSs in monocots. Whereas, the expression level of *HcTPS13* was constitutive in the plant (Figs. 4 and 6), indicating other unknown roles in *H. coronarium*. In higher plants, emission of volatile terpenes is often regulated at the transcriptional level of *TPS* genes [9]. For example, in snapdragon flowers, the emission of (*E*)-β-ocimene and myrcene closely correlate with the specific expression patterns of their corresponding *TPS* genes during flower development [15]. In *H. coronarium*, HcTPS7 and HcTPS8 have been characterized as sabinene synthase and linalool synthase, which involved in the formation of floral sabinene and linalool, respectively. However, individual HcTPS8 could not account for the total linalool production [46]. The transcript level of plastidial HcTPS1, HcTPS3 and HcTPS5 showed positive correlation with the monoterpenes emission, suggesting all these enzymes might be responsible for the generation of volatile monoterpenes. Meanwhile, cytosolic HcTPS4 and HcTPS10 possessing similar trends with the emission of sesquiterpenes, possibly account for the biosynthesis volatile sesquiterpene. Phylogenetic analysis showed that HcTPS6 belonged to TPS-b subfamily (Fig. 5b), which predominantly consists of angiosperm monoterpene synthases. However, it did not possess a plastidial signal peptide. Enzymatic activity analysis revealed that HcTPS6 was a bifunctional enzyme that could utilize GPP and FPP as substrates. Therefore, it is likely that HcTPS6 evolved from a parental monoterpene synthase through the loss of plastidial signal peptide. The similar event was also found in lavender. Lavender LaBERS in TPS-b clade, converts FPP into α-bergamotene in cytosol [79].

Methyl benzoate is synthesized through shikimate, arogenate pathway and subsequent benzenoid biosynthesis. DAHPS represents the first committed enzyme and controls the overall carbon flux into the pathway [64]. Petunia contains two DAHPSs. RNA interference (RNAi) suppression revealed PhDAHP1 with high expression level in petal limb and tube rather than PhDAHP2 with low expression level, which suggested that PhDAHP1 is responsible for the floral volatile benzenoid/phenylpropanoid biosynthesis [80]. Similarly, *HcDAHPS1*, with a very high expression level, appears to regulate the carbon flux into the shikimate pathway. In petunia, the generation of benzoic acid has been reported to proceed via β-oxidative pathway and non β-oxidative pathway [22]. Recently, the enzymes involved in the β-oxidative pathway have been fully elucidated in petunia through stable RNAi approach. In this pathway, the peroxisomal PhCNL is responsible for the first committed step [23, 26]. The *PhCNL* transcript was not detected in a closed flower bud while was initially detected at considerable levels at flowering stage and finally reduced in the end of the flower life-cycle [23, 26]. In addition, Arabidopsis BZO1, a member of AAE superfamily, has recently been confirmed to be responsible for the generation of cinnamonyl-CoA in β-oxidative pathway during seed development through knockout mutants [27]. Phylogenetic analysis revealed that HcCNL together with PhCNL and AtBZO1 fell into the group of PhCNL orthologs in clade VI AAEs (Fig. 9a). However, the CNLs in dicots and monocots cluster in two separate monophyletic branches and diverge from other AAE clades (Fig. 9a), indicating they evolved from the same ancestral CNL that exists before the split of dicots and monocots. Like *PhCNL*, the expression pattern of *HcCNL* exhibited positive correlation with levels of produced benzenoid compounds (Fig. 8), indicating that HcCNL might play a key role in supplying the carbon flux through β-oxidative pathway. In petunia, it was estimated that the flux via non-β-oxidative pathway with benzaldehyde as a key intermediate was approximately two-fold higher than the flux through β-oxidative pathway [22]. Benzaldehyde is one of the major compositions in the floral volatile profile of petunia [75], while no benzaldehyde is detected in the floral volatile profile of *H. coronarium*. Therefore, the β-oxidative pathway might be dominant for supplying the metabolic flux into volatile benzenoids in *H. coronarium*. BCMT, the final enzyme in the biosynthesis of methyl benzoate, was well characterized in dicots petunia and snapdragon [66, 67]. Phylogenetic analysis revealed that three HcBCMTs are closely related to monocot rice salicylic acid methyltransferase (SAMT) and maize anthranilic acid methyltransferase (AAMT), but distant from other characterized BAMTs and BSMTs in dicots (Fig. 9b). This might be explained by the facts that the enzymes in this group have broad substrate specificity and that the enzymes in this group catalyzing different substrates in species with closer affinity are more similar to each other than enzymes with same functions in distantly related species [81]. Given that only the expression of *HcBCMT1* remains at high levels and shows a positively correlation with the emission of methyl benzoate (Fig. 1g and Fig. 8), *HcBCMT1* seems to be primarily responsible for the biosynthesis of methyl benzoate.

The total of 1,741 TFs was identified in *H. coronarium* transcriptome, exceeding the amount of 1,533 TFs estimated in the Arabidopsis genome [68]. Like in Arabidopsis, MYB, AP2-EREBP and bHLH represent the most abundant TF families. TFs play a paramount role in regulating expression of genes involved in secondary metabolism [69], including floral scent production. To date, only a few TFs regulating the expression of scent-related genes have been identified in model plants, four MYB members in petunia [29, 30, 32, 33] and one bHLH member in Arabidopsis [18]. We identified 454 TFs that showed differential expression during petal development. Among them, 143 TFs show positive correlations with the emission of volatile compounds, and predominantly distribute in MYB, AP2-EREBP, NAC, WRKY, bHLH TF families (Fig. 11). Thirteen TFs in MYB family were first investigated, and six R2R3-MYBs were identified. Q-PCR analysis revealed that two *HcMYBs* were flower-specific expression (Fig. 12), further supporting their possible involvement in floral scent production. Phylogenetic analysis showed that both *HcMYBs* did not fall into the same MYB subgroups with any one of known scent-related R2R3-MYB factors (data not shown), suggesting possible novel scent-related TFs. However, to get more powerful evidence for involvement of TFs in floral scent formation, we will carry out further experimental verification through molecular biological and genetic methods, such as yeast one-hybrid and trans-activation assays using promoters of key structural genes.

Nowadays, *H. coronarium* is cultivated as an ornamental plant in many tropical and subtropical regions because of its flower possessing intense and inviting fragrance and elegant shape. The flowers exhibit nocturnal anthesis and are pollinated by moth at night [82]. As expected, the flowers release high amount of floral volatiles when blooming (Fig. 1). The coincidence of intense emission timing of floral scent and period of moth activity indicates the pollinator-attracting role of floral fragrance in this species. Indeed, the nocturnal emission peak of floral scent components coinciding with the activity patterns of moth is a feature of moth-pollinated flowers, such as *Petunia hybrida* cv. Mitchell [75]*.* Recent research demonstrated that a floral bouquet not only consists of attractive compounds to floral pollinator, but also contains volatile compounds that function as defense against florivores or pathogens [83, 84]. It is likely that benzenoids have evolved as pollinator attracting signals, while terpenoids serve as defensive compounds [83, 85, 86]. The feeding trials showed that the artificial diets adding linalool and β-caryophyllene represent effective antifeedants for *Metrioptera bicolor*, a bush cricket that occasionally feeds on flowers [86]. In addition, (*E*)-β-caryophyllene emitted from stigmas of Arabidopsis flowers was shown to act through direct inhibition of bacterial growth [84]. Interestingly, unlike other volatiles, the emission of 1,8-cineole and β-ocimene in *H. coronarium* increased continuously during flower development (Fig. 1), implying their possibly role in protecting pollinated flower tissues from florivores or pathogens.

## Conclusions

Using RNA-Seq technology, we first obtained *H. coronarium* transcriptome data that would provide an important resource for functional genomics studies. The DGE profile data gave us a dynamic view of biological process during petal development. Moreover, combination of floral volatile profiling and DGE profiling discovered several candidate scent-related genes in this monocot. Among them, flower-specific HcDXS2A, HcGPPS and HcTPSs might play critical roles in regulating the biosynthesis of the floral volatile terpenes, while flower-specific HcCNL and HcCBMT1 might function in controlling the formation of floral volatile methyl benzoate. HcTPS6 was characterized as β-farnesene synthase. We also identified and classified all TFs in the *H. coronarium* transcriptome. Wherein, two R2R3-MYBs were flower-specific and developmental expression. These results provide useful information to elucidate the molecular mechanism of floral scent formation and regulation in *H. coronarium*. Meanwhile, the results also provide the opportunities for breeding and genetic manipulation of scent-associated traits in *Hedychium* to enhance commercial values.

## Methods

### Plant material

*H. coronarium* was grown in the horticulture chamber in South China Agricultural University under natural light (Guangzhou, China). The materials used for RNA-Seq and Q-PCR were collected in September and were stored at −80 °C after immediately frozen in liquid nitrogen. The materials used for volatiles analysis were bought from a local *H. coronarium* cut flowers farm. The cut flowers were immediately cultured in MS liquid medium after they were brought back to laboratory. They had the similar flower developmental process with natural flowers.

### Headspace collection and GC-MS analysis

The flower developmental process from squaring stage to senescence stage was divided into six stages starting at 10:00 AM with 12-h intervals (Fig. 1a). The headspace collection and GC-MS analysis were performed as described previously [46]. The whole flower of each stage was enclosed in a 500-ml glass bottle with the addition of 1.728 μg ethyl caprate as internal standard. After equilibrium of volatiles for 30 min, a polydimethylsiloxane (PDMS, with 50/30 μm divinylbenzene/Carboxen) fiber (Supelco) was inserted into the bottle to adsorb volatiles for 30 min. Then, trapped floral scent compounds were analyzed by a GC-MS system with Agilent 7890A GC and Agilent 5975C MSD. The instrument was equipped with an Agilent HP-5MS capillary column (30 m × 0.25 mm) and helium as a carrier gas at a constant flow of 1 ml/min. The oven temperature was initially maintained at 40 °C for 2 min, followed by an increase to 250 °C at a rate of 5 °C/min, and held at 250 °C for 5 min. The volatiles were identified by comparing the mass spectra and retention times with authentic standards. Quantification was based on peak areas and the quantity of internal standard using Agilent ChemStation Data Analysis Application. Analysis of variance was performed by SPSS software using Duncan test (*P* = 0.05).

### RNA extraction and sequencing

For transcriptome assembly, the whole flowers with six biological replicates were sampled at stage D1, D4 and D6, respectively (Fig. 1a). The leaves and rhizomes from three biological replicates were sampled at D4. Total RNA from flowers at three different developmental stages was isolated independently with RNAiso Plus reagent (TaKaRa) following the manufacturer’s protocol. Total RNA of leaves and rhizomes were isolated using the improved hexadecyl trimethyl ammonium bromide (CTAB) method as described previously [46]. The transcriptome sequencing library was pooled by mixing equal quantities of RNA from flowers (three flower developmental stages), leaves and rhizomes. For DGE analysis, petals with ten biological replicates were harvested from three abovementioned developmental stages, respectively. Total RNA was isolated independently with RNAiso Plus reagent as described above. Total RNA purity and degradation was detected on 1 % agarose gels and NanoPhotometer spectrophotometer (IMPLEN). RNA concentration and integrity was measured with a Qubit 2.0 Fluorometer (Life Technologies) and an Agilent 2100 Bioanalyzer, respectively. Sequencing libraries for transcriptome assembly and DGE analysis were generated using Illumina TruSeq RNA Sample Preparation Kit according to the manufacturer’s instructions. Briefly, poly-(A) mRNA was isolated from total RNA using oligo-(T) magnetic beads. The purified mRNA was fragmented using divalent cations under elevated temperature. With mRNA fragmentation as template, first strand cDNA was synthesized using random hexamer-primers and SuperScript II. Second-stranded cDNA was synthesized using RNase H and DNA polymerase I. Subsequently, double-stranded cDNA fragments underwent end repair, adenylation of 3’ ends and ligation of Illumina PE adapter oligonucleotides. The adaptor modified fragments were purified with AMPure XP system (Beckman) to preferentially select 200 bp fragments. After that, suitable fragment were enriched using Illumina PCR Primer Cocktail in a 10 cycle PCR reaction. Products were purified with AMPure XP system and quantified using Agilent 2100 Bioanalyzer system. The clustering of the index-coded samples was performed on a cBot Cluster Generation System using TruSeq PE Cluster Kit v3-cBot-HS (Illumia) following manufacturer’s recommendations. After cluster generation, the library preparations were sequenced on an Illumina Hiseq 2000 platform at Beijing Novogene Bioinformatics Technology Corporation. The 100 bp paired-end reads were generated for transcriptome assembly and 100 bp single-end reads were generated for DGE analysis. The transcriptome data and DGE profiling data were deposited in the NCBI Sequence Read Archive (SRP049915).

### *De novo* assembly and functional annotation

Raw reads were filtered to obtain high-quality clean reads by removing reads containing adapter, reads containing ambiguous “N” nucleotides (with the ratio of more than 10 %) and low quality reads containing more than 50 % bases with *Q*-value ≤ 5. All the following analyses were based on clean reads. Transcriptome assembly was performed using Trinity software [35] with min_kmer_cov set to 2 and all other parameters set default as described for *de novo* assembly without reference genome. All assembled unigenes were used for blast search and annotation against public databases including NCBI Nr and Nt, Swiss-Prot, Pfam, GO, COG and KEGG databases. Based on sequence similarity, assembled unigenes were compared to Nr, Nt and Swiss-Prot databases using BLASTX algorithm with a signification threshold of *E*-value ≤10^−5^ (≤10^−10^ for Nr). Pfam protein database was searched against using HMMER 3.0 program [87] with an E-value threshold of 10^−5^. Functional categorization by GO terms was performed using Blast2GO software [88]. KOG and KEGG pathway annotation were carried out using BLASTX algorithm with an E-value cut-off of 10^−5^.

### Quantification of gene expression levels

Prior to gene quantification, raw reads from three developmental stages were filtered to obtain high-quality clean reads as described above. For gene expression analysis, clean reads were mapped to assembled reference transcriptome, and then readcount of each gene was estimated using RSEM [89] in each library. Subsequently, readcount was normalized to RPKM [50]. The RPKM method considering the effect of gene length and sequencing depth on the calculation of gene expression is commonly used for estimating gene expression levels.

### Differential expression analysis

Differential expression analysis between two samples was performed using the DEGseq R package [90]. *P* value was adjusted using *q* value [91]. The absolute value of log_2_(fold change) > 1 and *q* value < 0.005 was set as the threshold to judge the significant DEGs. GO term enrichment analysis of DEGs was implemented by GOseq R package based on Wallenius non-central hyper-geometric distribution [92], which can adjust gene length bias. KEGG pathway enrichment analysis of the DEGs was performed using KOBAS software [93].

### Analysis of genes involved in the formation of floral scent

Since the floral volatiles of *H.coronarium* fall into the classes of terpene and phenylpropanoid, genes encoding enzymes involved in the biosynthesis pathway of terpene and phenylpropanoid were analyzed. The biosynthesis pathway of terpene and phenylpropanoid were plotted referring to the KEGG pathway and some literatures. The genes related to the metabolism were mined according to the KEGG annotation and local blast search with an E-value threshold of 10^−5^. The query sequences used for local blast search were the corresponding homologous gene functionally characterized in other plants. For larger-size gene family searched by local blast, phylogenetic analysis was done to distinguish the orthologs of corresponding functionally characterized genes. The phylogenetic tree was constructed with MEGA4 after the amino acid sequences were aligned using ClustalX. Subcellular localization analysis was performed using TargetP 1.1 Server (http://www.cbs.dtu.dk/services/TargetP/) and WoLF PSORT (http://www.genscript.com/psort/wolf_psort.html).

### Q-PCR

The primers for Q-PCR were designed in the 3′ untranslated or other specific regions with Primer 5.0 software based on the assembled transcriptome sequences. The primers sequences are listed in Additional file 14. One microgram of total RNA was reverse transcribed using the PrimeScript RT reagent Kit with gDNA Eraser (TaKaRa) according to the manufacturer’s instructions. Q-PCR was performed in a volume of 20 μl containing iTaq Universal SYBR Green Supermix (Bio-Rad), cDNA solution, forward and reverse primers following the manufacturer’s recommendations, with an ABI 7500 Real-Time PCR System. The PCR conditions were as follows: 95 °C for 1 min, followed by 40 cycles of 95 °C for 15 s, 55 °C for 30 s, 72 °C for 30 s. Three independent amplifications were performed for each sample. Previous validated genes *RPS* and *ACT* were used as reference genes at different petal developmental stages and in different organs, respectively [46]. The specificity of each primer pair was verified by agarose gel electrophoresis analysis and melting curve analysis. The relative expression levels of target genes were calculated by 2^-ΔΔCt^ method [94]. The error bars represent the calculated maximum (2^-(ΔΔCt - SE)^) and minimum (2^-(ΔΔCt + SE)^) expression quantity, where SE is the standard error of the ΔΔCt value. Analysis of variance was performed by SPSS software using Duncan test (*P* = 0.05).

### Heterologous expression of HcTPS6 and enzyme assay

The coding region of HcTPS6 was amplified using high-fidelity DNA polymerase KOD-Plus (TOYOBO) with the forward primer 5′-*GGATCC*ATGGCTACTCGTCAAGCAAT-3′ and the reverse primer 5′-*GTCGAC*GGATTTGGATAGGTTCAAAT-3′, then cloned into pET30a vector (Novagen) through *Bam*HI and *Sal*I sites. The resulting constructs were sequenced for no errors and transformed into *E. coli* Rosetta (DE3) competent cells (Invitrogen). The induction, purified and concentration determination of recombinant protein were performed as described previously [46]. Enzyme assay was carried out in 5-ml sealed glass vials in a total volume of 1 ml containing 30 mM HEPES (pH 7.5), 5 mM DTT, 20 mM MgCl_2_, 20 μM GPP/FPP (Sigma) and ~10 μg recombinant HcTPS6 proteins. A PDMS fiber for solid-phase microextraction was inserted into the vial to collect volatiles. The mixture was incubated at 30 °C for 1 h and then at 45 °C for 15 min. After incubation, the PDMS fiber was injected into a gas chromatography–mass spectrometry (GC-MS) system for analysis as described above. The products were identified by comparing the mass spectra and retention times with authentic standards or known profiles of *H. coronarium*, or by comparing mass spectra with the NIST 08 mass spectra library.

### Analysis of transcription factors

Transcription factors were identified and classified using iTAK (http://bioinfo.bti.cornell.edu/cgi-bin/itak/index.cgi) based on protein domains of unigenes. Subsequently, the differentially expressed TFs were picked out from DEGs in D1 VS D4 or D4 VS D6, or both. STEM (Short Time-series Expression Miner) software (http://www.cs.cmu.edu/~jernst/stem/) was used to cluster the differentially expressed TFs and distinguish the temporal expression profiles.

## Additional files

Additional file 1:
**Length distribution of assembled unigenes.**
Additional file 2:
**Gene ontology (GO) classification of unigenes.** The GO terms are summarized into three main categories: biological process, cellular component and molecular function. Additional file 3:
**KOG classification of the unigenes.** A total of 13,240 unigenes were annotated against KOG database and assigned among the 26 functional categories. Additional file 4:
**KEGG classification of 5,933 KO annotated unigenes.**
Additional file 5:
**Summary of unigenes involved to secondary metabolism.**
Additional file 6:
**Uniform distribution of reads on reference genes of three libraries.**
Additional file 7:
**Saturation curves analysis of three libraries.** If the curve reach a plateau before 100 % mapped reads were used, this indicates that the gene group was sequenced exhaustively, as obtaining more reads does not change their RPKM. Additional file 8:
**GO terms significantly enriched during petal development.** Go terms with corrected p-value less than 0.05 are shown. Additional file 9:
**Enriched KEGG pathways during petal development.** Pathways with corrected p-value less than 0.05 are defined as significantly enriched terms. Additional file 10:
**Phylogenetic tree of HcTPS13 with other plant TPSs based on the neighbor-joining method.** Gymnosperm-specific TPS-d subfamily was further divided into TPS-d1 (primarily mono-TPS), TPS-d2 (sesqui-TPS) and TPS-d3 (primarily di-TPS). HcTPS13 and four closely related *Phoenix dactylifera* TPSs are shadowed in blue. Additional file 11:
**Alignment of deduced amino acid sequences of HcTPS13, HcTPS8 and four** ***Phoenix dactylifera*** **TPSs**. The HcTPS13 sequence contains the DDXXD motif, not DXDD motif. Additional file 12:
**Mass spectra of peaks in Figure** 6 **and their corresponding NIST08 standards.**
Additional file 13:
**Q-PCR validation of selected transcripts expression among three petal developmental stages.** Expression levels of selected transcripts measured by Q-PCR and RNA-Seq are showed in the same histograms. Additional file 14:
**Primers used in Q-PCR validation.**

## Abbreviations

AACT: Acetyl-CoA acetyltransferase
ADT: Arogenate dehydratase
BALD: Benzaldehyde dehydrogenase
BAMT: Benzoic acid carboxyl methyltransferase
BCMT: Benzenoid carboxyl methyltransferase
BSMT: Benzoic acid/salicylic acid carboxyl methyltransferases
CDS: Coding sequence
CHD: Cinnamoyl-CoA hydratase-dehydrogenase
CM: Chorismate mutase
CMK: 4-(cytidine 5′-diphospho)-2-C-methyl-D-erythritol kinase
CNL/AAE: Cinnamate:CoA ligase/acyl-activating enzyme
CS: Chorismate synthase
DAHPS: 3-Deoxy-7-phosphoheptulonate synthase
DEG: Differentially expressed gene
DGE: Digital gene expression
DHD/SHD: dehydroquinate dehydratase/shikimate dehydrogenase
DHQS: 3-Dehydroquinate synthase
DMAPP: Dimethylallyl pyrophosphate
DXS: 1-Deoxy-D-xylulose 5-phosphate synthase
DXS: 1-Deoxy-D-xylulose 5-phosphate synthase
E4P: Erythrose 4-phosphate
EPSPS: 3-Phospho shikimate 1-carboxyvinyltransferase
EST: Expressed sequence tag
FPP: Farnesyl diphosphate
FPPS: FPP synthase
GC-MS: Gas chromatograph-mass spectrometer
GGPP: Geranylgeranyl diphosphate
GGPPS: GGPP synthase
GO: Gene Ontology
GPP: Geranyl diphosphate
GPPS: GPP synthase
HDR: 4-Hydroxy-3-methylbut-2-en-1-yl diphosphate reductase
HDS: 4-Hydroxy-3-methylbut-2-en-1-yl diphosphate synthase
HMGR: Hydroxymethylglutaryl-CoA reductase
HMGS: Hydroxymethylglutaryl-CoA synthase
IDI: Isopentenyl diphosphate isomerase
IPP: Isopentenyl pyrophosphate
KAT: 3-Ketoacyl CoA thiolase
KEGG: Kyoto Encyclopedia of Genes and Genomes
KO: KEGG orthology
KOG: euKaryotic Ortholog Groups
MCT: 2-C-methyl-D-erythritol 4-phosphate cytidylyltransferase
MDC: Mevalonate diphosphate decarboxylase
MDS: 2-C-methyl-D-erythritol-2,4-cyclodiphosphate synthase
MEP: 2-C-methyl-D-erythritol 4-phosphate
MK: Mevalonate kinase
MVA: Mevalonate
Nr: NCBI non-redundant protein
Nt: NCBI non-redundant nucleotide
PAL: Phenylalanine ammonialyase
PAT: Prephenate aminotransferase
PDMS: Polydimethylsiloxane
PEP: Phosphoenolpyruvate
Pfam: Protein family
PMK: Phosphomevalonate kinase
PXA: Peroxisomal ATP-binding cassette transporter
Q-PCR: Real-time quantitative PCR
RPKM: Reads per kilobase of exon model per million mapped reads
SAM: *S*-adenosyl-L-methionine
SK: Shikimate kinase
TF: Transcription factors
TPS: Terpene synthase
