# Supplementary figures and images for: Transcriptome profiling provides new insights into the formation of floral scent in Hedychium coronarium
Source: BMC Genomics. 2015 Jun 19;16(1):470. doi: 10.1186/s12864-015-1653-7 (PMC4472261; doi:10.1186/s12864-015-1653-7)

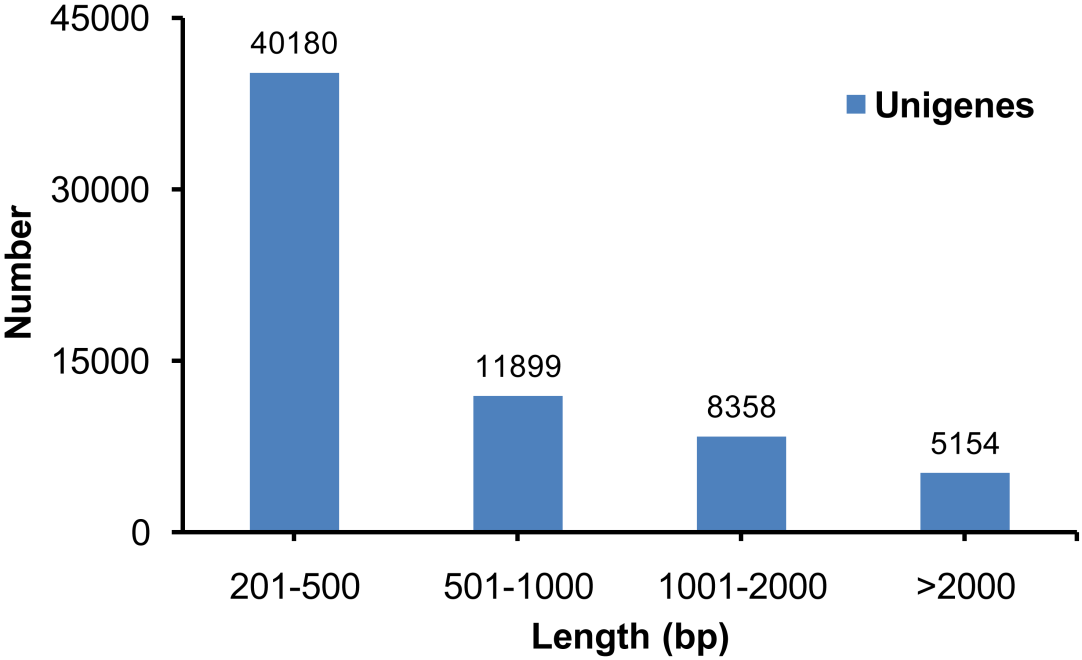


**Length distribution of assembled unigenes.**

Supplement: Additional file 1: — Length distribution of assembled unigenes. [file 12864_2015_1653_MOESM1_ESM.docx]

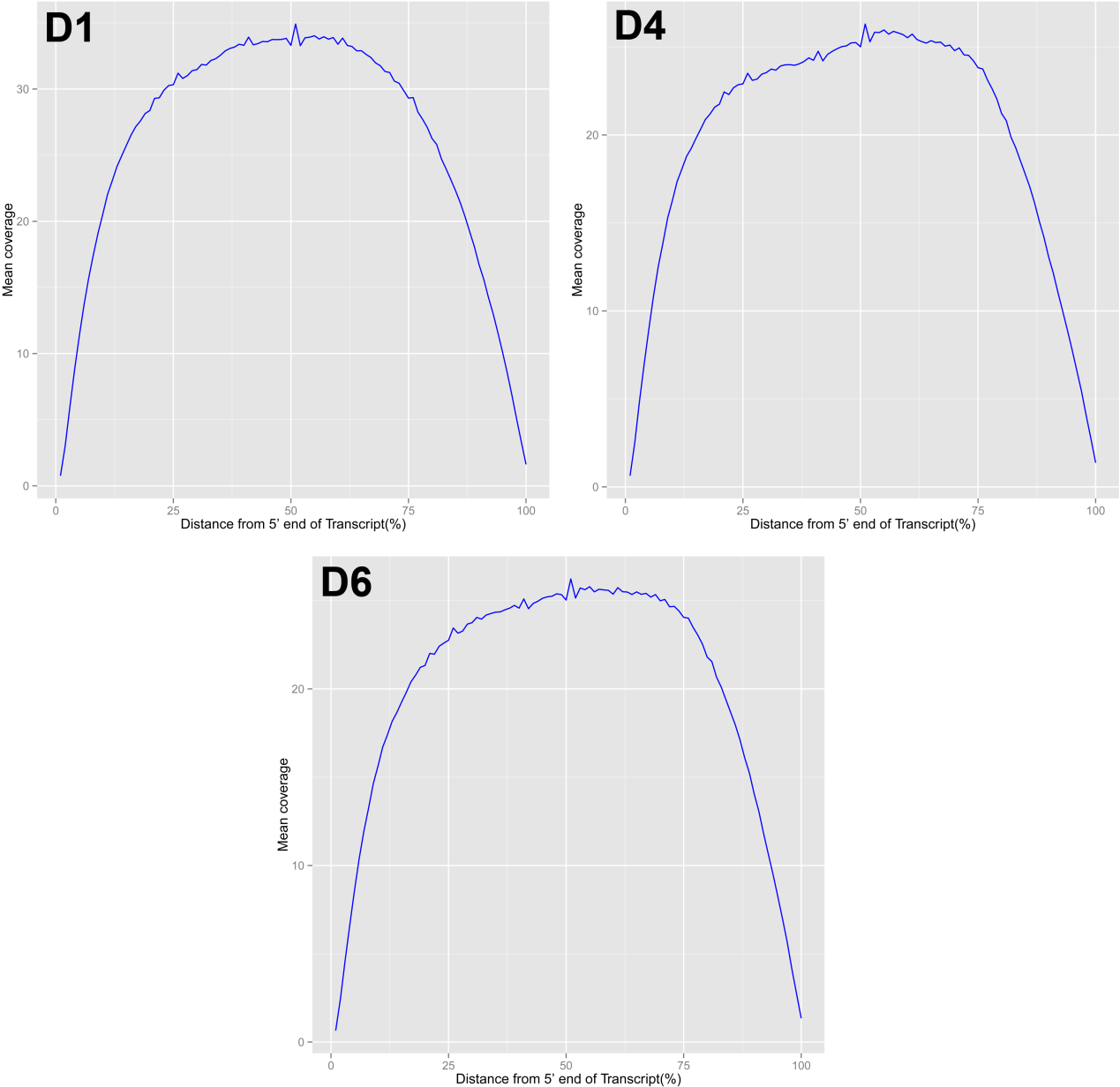


**Uniform distribution of reads on reference genes of three libraries.**

Supplement: Additional file 6: — Uniform distribution of reads on reference genes of three libraries. [file 12864_2015_1653_MOESM6_ESM.docx]

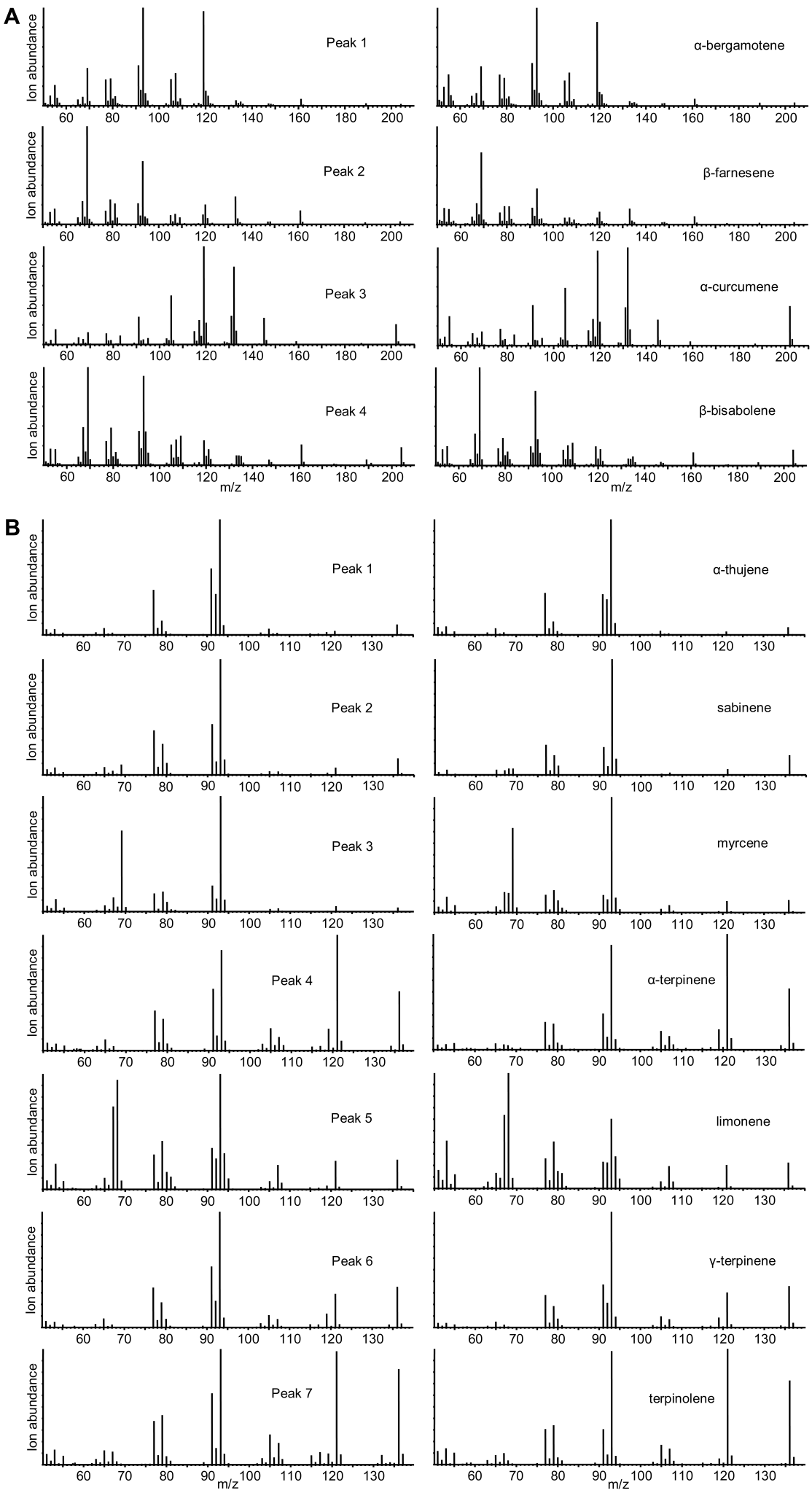


**Mass spectra of peaks in Figure 6 and their corresponding NIST08 standards.**

Supplement: Additional file 12: — Mass spectra of peaks in Figure 6 and their corresponding NIST08 standards. [file 12864_2015_1653_MOESM12_ESM.docx]
