# Supplementary material for: Transcriptome profiling provides new insights into the formation of floral scent in Hedychium coronarium
Source: BMC Genomics. 2015 Jun 19;16(1):470. doi: 10.1186/s12864-015-1653-7 (PMC4472261; doi:10.1186/s12864-015-1653-7)
Supplement: Additional file 2: — Gene ontology (GO) classification of unigenes. The GO terms are summarized into three main categories: biological process, cellular component and molecular function. [file 12864_2015_1653_MOESM2_ESM.docx]

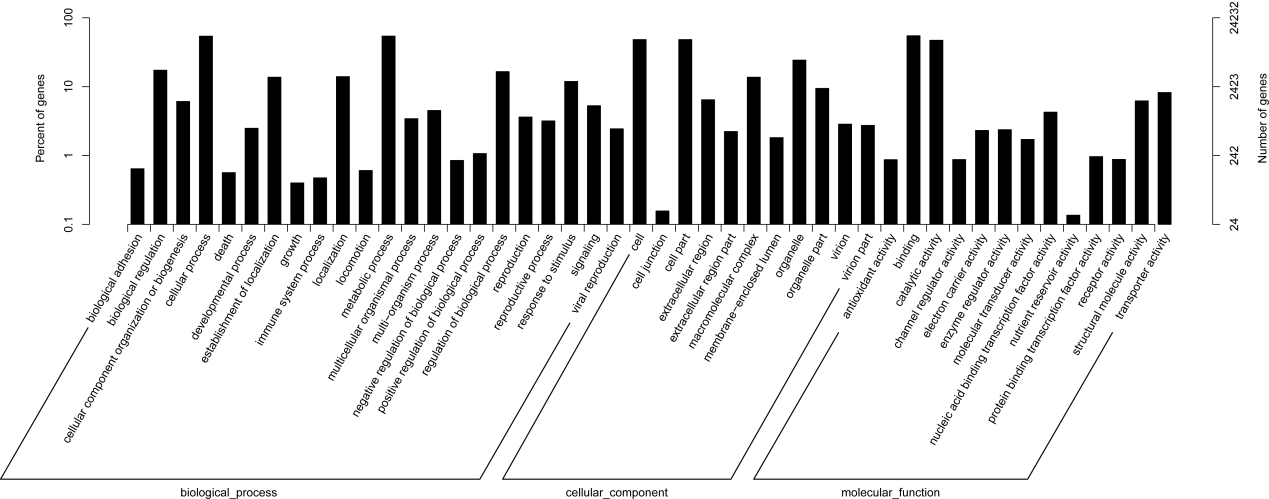


**Gene ontology (GO) classification of unigenes.** The GO terms are summarized into three main categories: biological process, cellular component and molecular function. The left y-axis indicates the percentage of a specific category of genes in that main category. The right y-axis indicates the number of annotated unigenes in a category.
