# Supplementary material for: Transcriptome profiling provides new insights into the formation of floral scent in Hedychium coronarium
Source: BMC Genomics. 2015 Jun 19;16(1):470. doi: 10.1186/s12864-015-1653-7 (PMC4472261; doi:10.1186/s12864-015-1653-7)
Supplement: Additional file 3: — KOG classification of the unigenes. A total of 13,240 unigenes were annotated against KOG database and assigned among the 26 functional categories. [file 12864_2015_1653_MOESM3_ESM.docx]

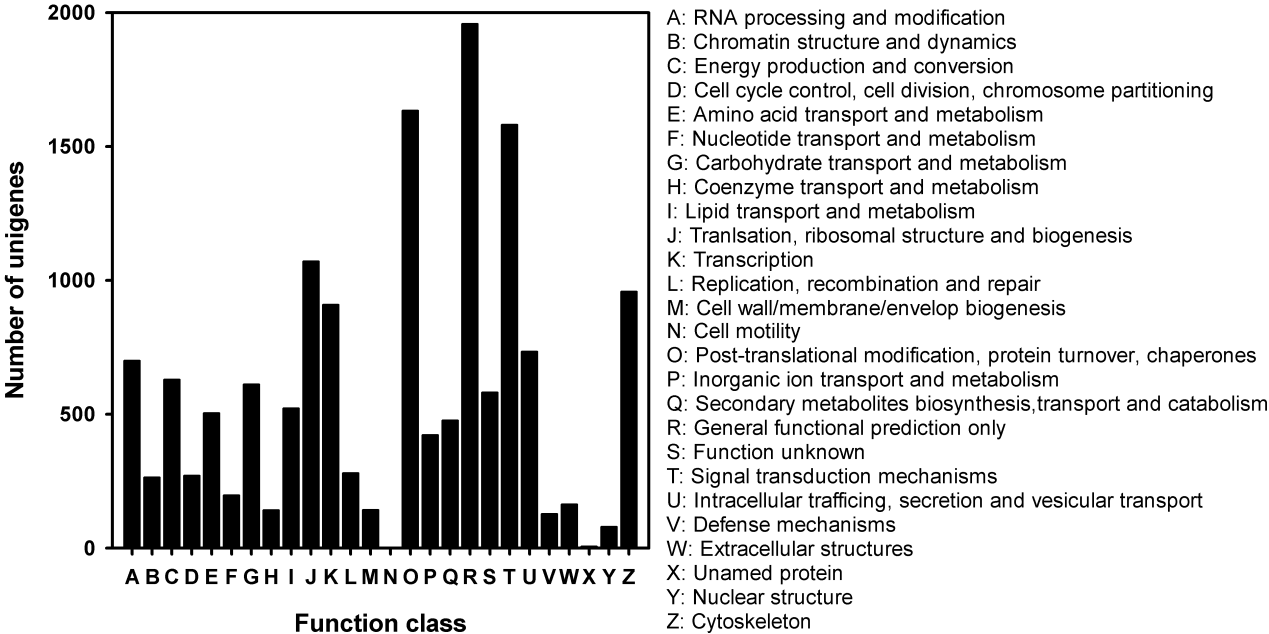


**KOG classification of the unigenes.** A total of 13,240 unigenes were annotated against KOG database and assigned among the 26 functional categories
