# Supplementary material for: Transcriptome profiling provides new insights into the formation of floral scent in Hedychium coronarium
Source: BMC Genomics. 2015 Jun 19;16(1):470. doi: 10.1186/s12864-015-1653-7 (PMC4472261; doi:10.1186/s12864-015-1653-7)
Supplement: Additional file 5: — Summary of unigenes involved to secondary metabolism. [file 12864_2015_1653_MOESM5_ESM.docx]

**Summary of unigenes involved to secondary metabolism**

| **Pathway of secondary metabolism** | **Number of unigenes** |
| --- | --- |
| Anthocyanin biosynthesis | 2 |
| Brassinosteroid biosynthesis | 14 |
| Caffeine metabolism | 4 |
| Carotenoid biosynthesis | 35 |
| Diterpenoid biosynthesis | 18 |
| Flavone and flavonol biosynthesis | 13 |
| Flavonoid biosynthesis | 43 |
| Isoquinoline alkaloid biosynthesis | 12 |
| Limonene and pinene degradation | 17 |
| Monoterpenoid biosynthesis | 2 |
| Phenylpropanoid biosynthesis | 109 |
| Sesquiterpenoid and triterpenoid biosynthesis | 7 |
| Stilbenoid, diarylheptanoid and gingerol biosynthesis | 18 |
| Terpenoid backbone biosynthesis | 80 |
| Tropane, piperidine and pyridine alkaloid biosynthesis | 20 |
| Zeatin biosynthesis | 13 |
