# Supplementary material for: Transcriptome profiling provides new insights into the formation of floral scent in Hedychium coronarium
Source: BMC Genomics. 2015 Jun 19;16(1):470. doi: 10.1186/s12864-015-1653-7 (PMC4472261; doi:10.1186/s12864-015-1653-7)
Supplement: Additional file 7: — Saturation curves analysis of three libraries. If the curve reach a plateau before 100 % mapped reads were used, this indicates that the gene group was sequenced exhaustively, as obtaining more reads does not change their RPKM. [file 12864_2015_1653_MOESM7_ESM.docx]

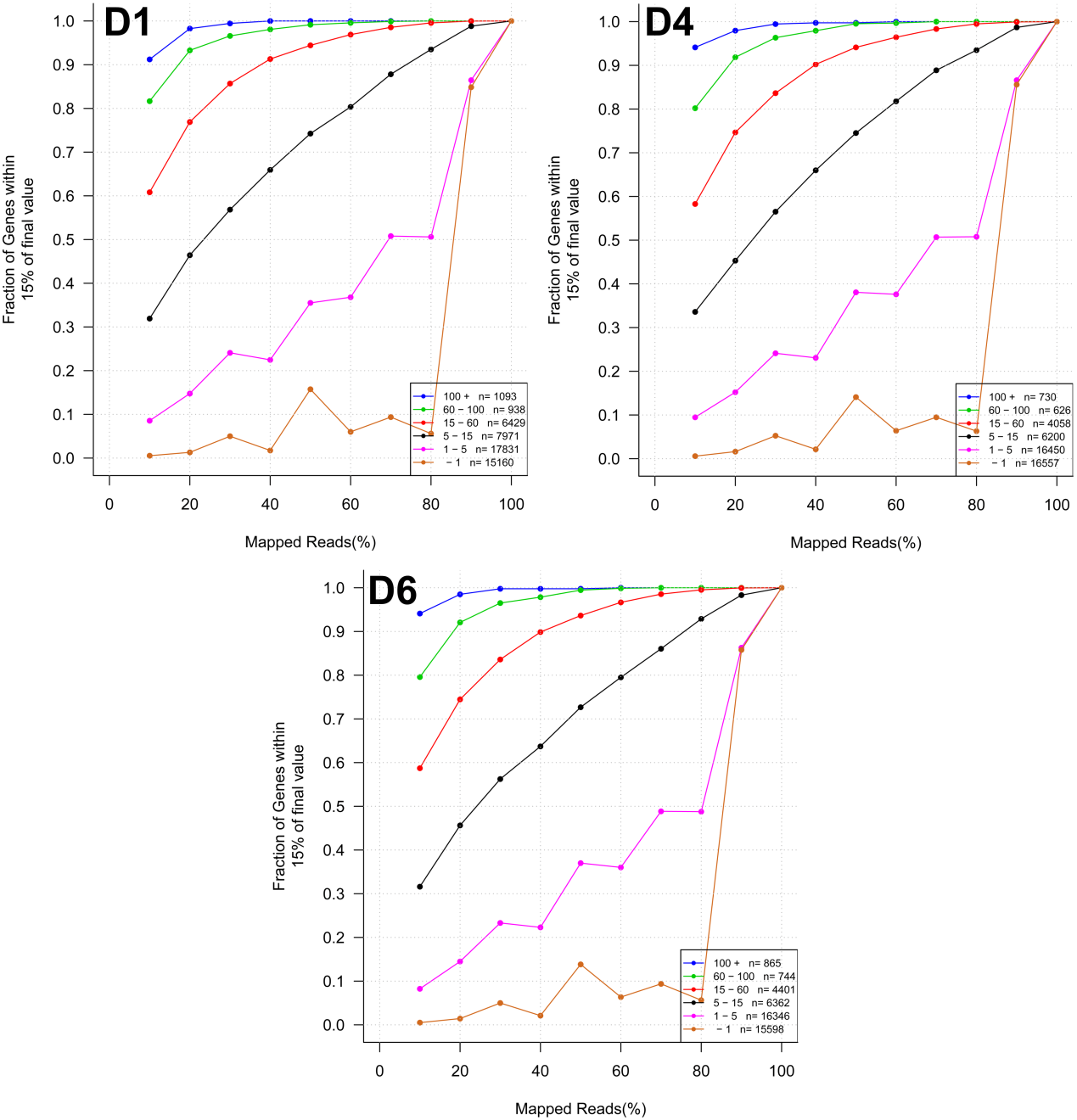


**Saturation curves analysis.** The y-axis indicates the fraction of genes number with RPKM value within 15% error of the final value. The x-axis indicates the percentage of mapped reads used for quantification. Curves in different colors represent different RPKM intervals calculated with 100% mapped reads. “n” represent the gene number in the corresponding RPKM interval. If the curve reach a plateau before 100% mapped reads were used, this indicates that the gene group was sequenced exhaustively, as obtaining more reads does not change their RPKM.
