# Supplementary material for: Transcriptome profiling provides new insights into the formation of floral scent in Hedychium coronarium
Source: BMC Genomics. 2015 Jun 19;16(1):470. doi: 10.1186/s12864-015-1653-7 (PMC4472261; doi:10.1186/s12864-015-1653-7)
Supplement: Additional file 10: — Phylogenetic tree of HcTPS13 with other plant TPSs based on the neighbor-joining method. Gymnosperm-specific TPS-d subfamily was further divided into TPS-d1 (primarily mono-TPS), TPS-d2 (sesqui-TPS) and TPS-d3 (primarily di-TPS). HcTPS13 and four closely related Phoenix dactylifera TPSs are shadowed in blue. [file 12864_2015_1653_MOESM10_ESM.docx]

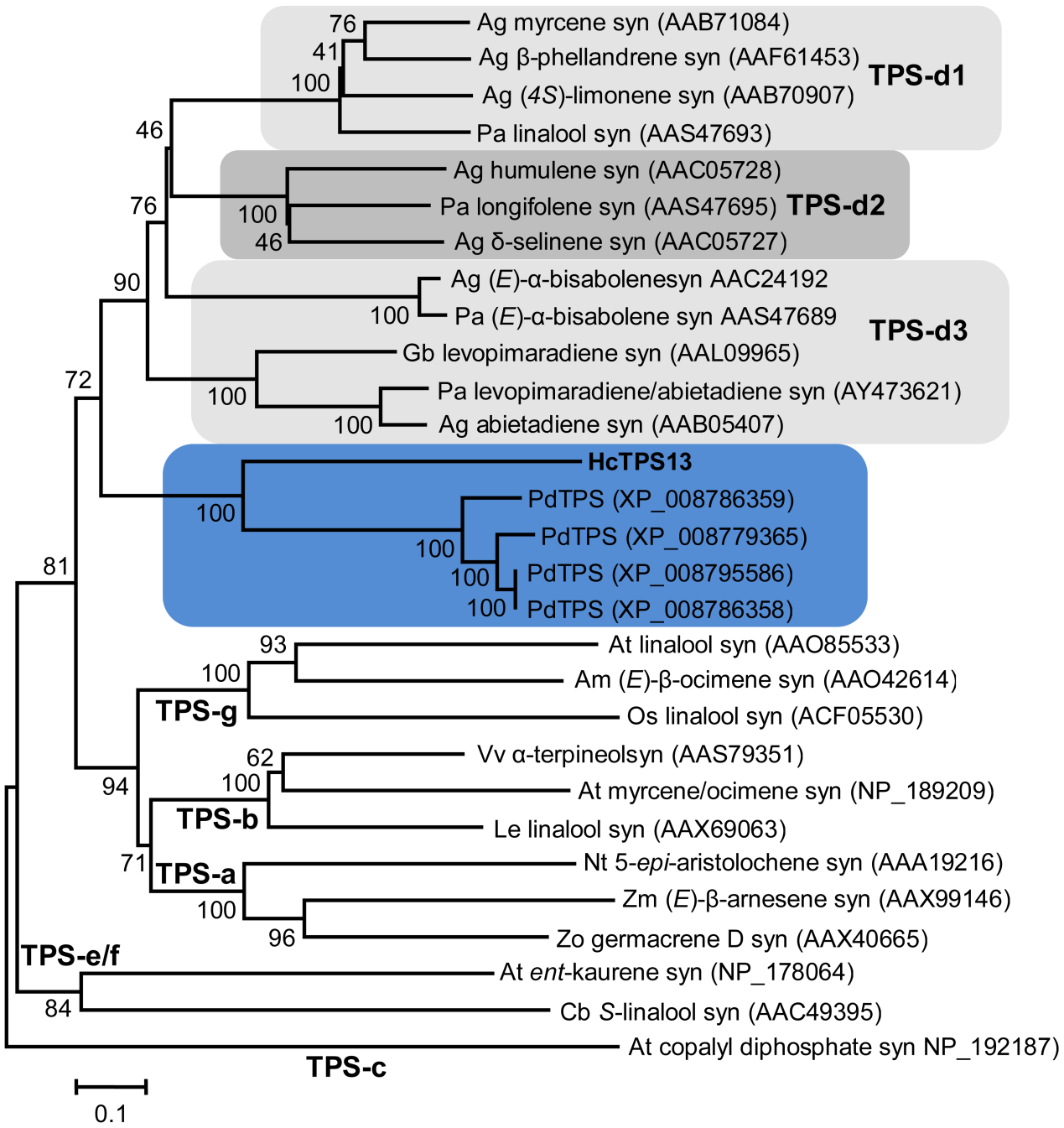


**Phylogenetic tree of HcTPS13 with other plant TPSs based on the neighbor-joining method.** Gymnosperm-specific TPS-d subfamily was further divided into TPS-d1 (primarily mono-TPS), TPS-d2 (sesqui-TPS) and TPS-d3 (primarily di-TPS). HcTPS13 and four closely related *Phoenix dactylifera* TPSs are shadowed in blue. The scale bar indicates 10% sequence divergence. The numbers at each branch indicate bootstrap percentages from 1000 replicates. GenBank accession numbers are shown in parentheses. Ag, *Abies grandis*; Am, *Antirrhinum majus*; At, *Arabidopsis thaliana*; Cb, *Clarkia breweri*; Gb, *Ginkgo biloba*; Le, *Lycopersicon esculentum*; Os, *Oryza sativa*; Pa, *Picea abies*; Pd, *P. dactylifera*; Nt, *Nicotiana tabacum*; Vv, *Vitis vinifera*; Zm, *Zea mays*; Zo, *Zingiber officinale*.
