# Supplementary material for: Transcriptome profiling provides new insights into the formation of floral scent in Hedychium coronarium
Source: BMC Genomics. 2015 Jun 19;16(1):470. doi: 10.1186/s12864-015-1653-7 (PMC4472261; doi:10.1186/s12864-015-1653-7)
Supplement: Additional file 11: — Alignment of deduced amino acid sequences of HcTPS13, HcTPS8 and four Phoenix dactylifera TPSs. The HcTPS13 sequence contains the DDXXD motif, not DXDD motif. [file 12864_2015_1653_MOESM11_ESM.docx]

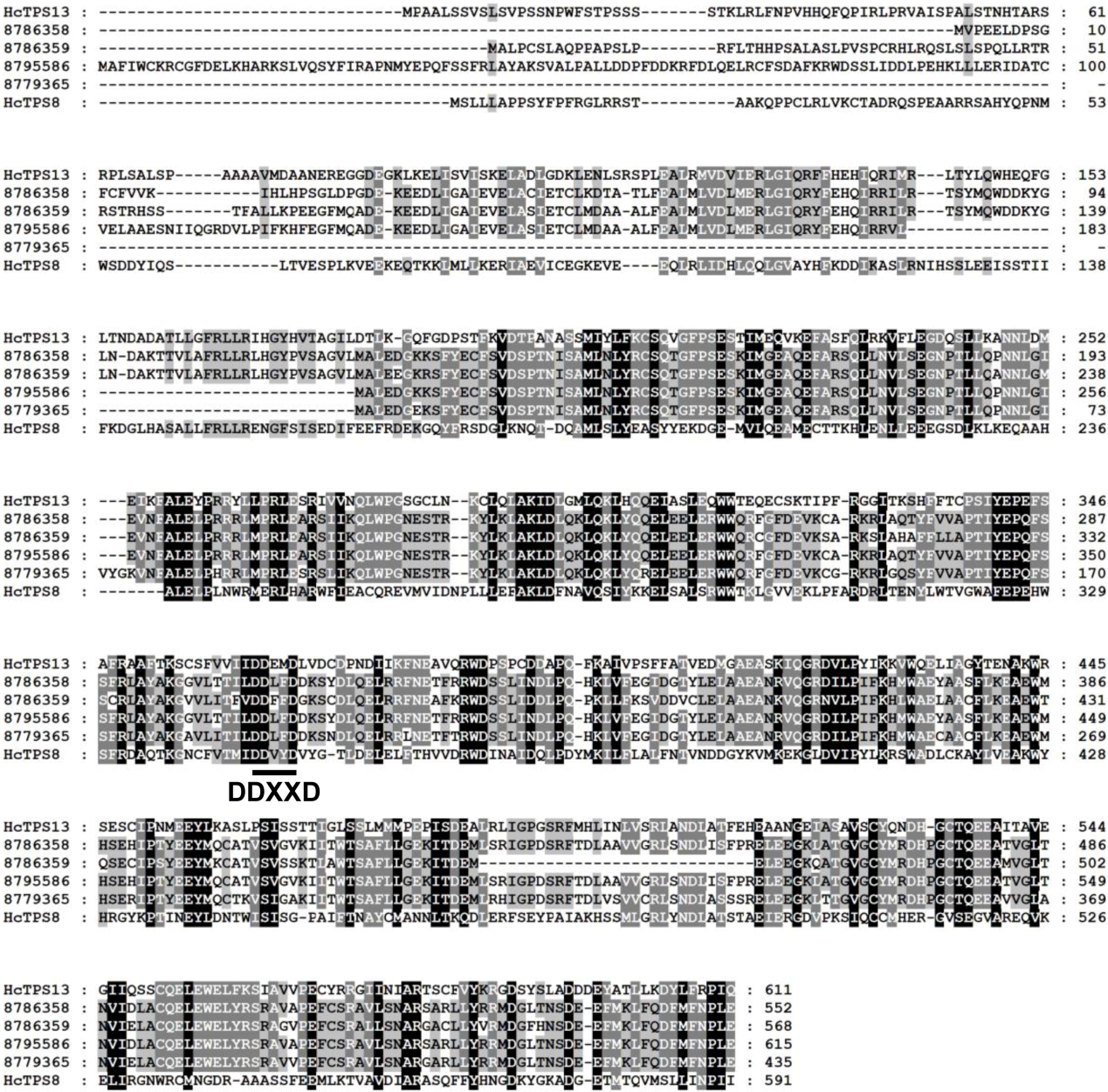


**Alignment of deduced amino acid sequences of HcTPS13, HcTPS8 and four *P. dactylifera* TPSs** (8786358: XP_008786358; 8786359: XP_008786359; 8795586: XP_008795586; 8779365: XP_008779365). Amino acid residues shaded in black, gray and light gray represent 100, 80 and 60 % conserved identity, respectively. Dashes indicate gaps inserted for optimal alignment. The conserved DDXXD motifs of TPSs are underlined.
