# Supplementary material for: Transcriptome profiling provides new insights into the formation of floral scent in Hedychium coronarium
Source: BMC Genomics. 2015 Jun 19;16(1):470. doi: 10.1186/s12864-015-1653-7 (PMC4472261; doi:10.1186/s12864-015-1653-7)
Supplement: Additional file 13: — Q-PCR validation of selected transcripts expression among three petal developmental stages. Expression levels of selected transcripts measured by Q-PCR and RNA-Seq are showed in the same histograms. [file 12864_2015_1653_MOESM13_ESM.docx]

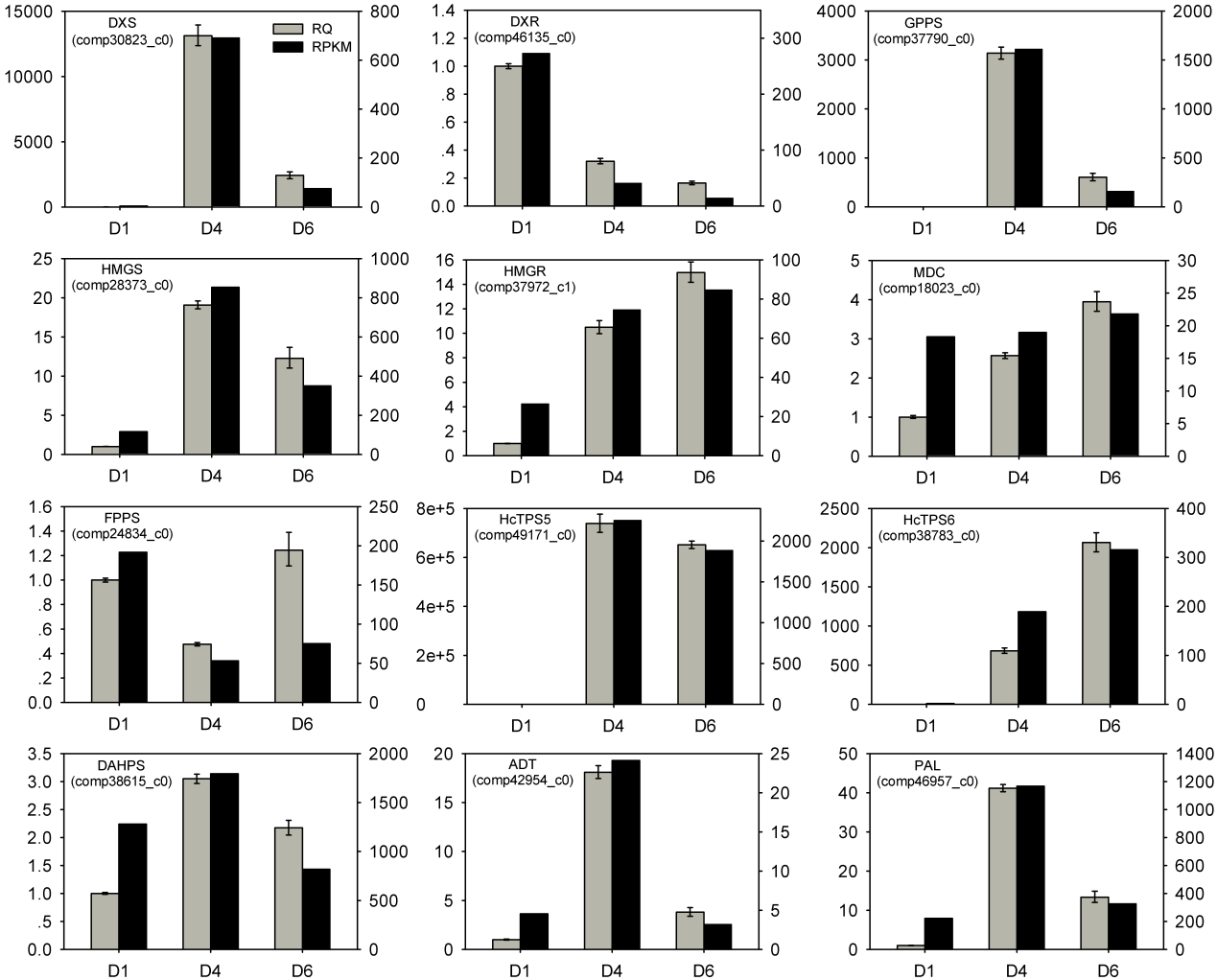


**Q-PCR validation of selected transcripts expression among three petal developmental stages.** Expression levels of selected transcripts measured by Q-PCR and RNA-Seq are showed in the same histograms. Grey columns indicate relative gene expression levels detected by Q-PCR (left y-axis; normalized units). Black columns represent expression determined by RNA-Seq in RPKM units (right y-axis).
