# Supplementary material for: Transcriptome profiling provides new insights into the formation of floral scent in Hedychium coronarium
Source: BMC Genomics. 2015 Jun 19;16(1):470. doi: 10.1186/s12864-015-1653-7 (PMC4472261; doi:10.1186/s12864-015-1653-7)
Supplement: Additional file 14: — Primers used in Q-PCR validation. [file 12864_2015_1653_MOESM14_ESM.docx]

**Primers used for Q-PCR**

| **Gene** | **Description** | **Forward primer (5′ to 3′)** | **Reverse primer (5′ to 3′)** |
| --- | --- | --- | --- |
| HcDXS2A | 1-Deoxy-D-xylulose 5-phosphate synthase | CCACTGAACCTACAAGAC | CTCATTACACTAACCCATAG |
| HcDXR | 1-Deoxy-D-xylulose 5-phosphate reductoisomerase | TTGAGTCCTGTGGCTGCTTG | GACCAGGGAGATGGGAATAAGT |
| HcGPPS | Geranyl pyrophosphate synthase | CTGATGGGGCTGGAGAAG | TAATTCAGTTGTGGCGGTTG |
| HcHMGS | Hydroxymethylglutaryl-CoA synthase | CTTACTGACACCTGGAACCT | TTCAGCAACAGCATCACCTG |
| HcHMGR | Hydroxymethylglutaryl-CoA reductase | TATCTGTGTGGGAGGTTGACTG | TGAACTCAGGAACATCAAACCG |
| HcMDC | Mevalonate diphosphate decarboxylase | ATTGTGGTTCGCTCGTCATCCT | ACAGGACTCAGACACCAGATAG |
| HcFPPS | Farnesyl pyrophosphate synthase | ATGAGCGGGCTAGTTATG | GATAGATTACTTCTGCCTCT |
| HcTPS1 | Terpene synthase | GCTCTACGACGATTTAGCAACTTC | CACGATCTCCATTGACGCACCT |
| HcTPS3 | Terpene synthase | TGATGGCTGCTCGGTCATTC | TTCATTCGATCCCCGTTCATGG |
| HcTPS4 | Terpene synthase | GGCTATACTAATTCCAACACTAGG | GGCATTGCCCTGATAAGACATAC |
| HcTPS5 | Terpene synthase | ATTACTTGCGGTGTTCCTGCTG | AACGAGCCATCCCTCCCATT |
| HcTPS6 | Terpene synthase | GCACAGTTCTTTTATGAGGATGACT | GATCCATATATCCTCCCGTCTAC |
| HcTPS10 | Terpene synthase | CCTTCATAATGCCAAACTGTTCTC | TACAAGCGATGTTCAAGGTGGTC |
| HcTPS13 | Terpene synthase | GAGCTGGAGTGGGAACTGTTC | GAATAGGGCGGAAGAGGTAATC |
| HcDAHPS | 3-Deoxy-7-phosphoheptulonate synthase | TTGGAGGTGTGGACTCACTATG | TCCATGCCAGAACAAGCTTAAC |
| HcADT | Arogenate dehydratase | GATGTGGATCTTCGAGAG | AAGACGTAGTCCAGGTAAGC |
| HcPAL | Phenylalanine ammonia lyase | CTCATGTTCGCCCAATTCTC | TTATGCTGCTCCGCACTCT |
| HcBCMT1 | Benzenoid carboxyl methyltransferase | AGGAGAAAGCCAATCACACC | GGCATGATTCAGTTTGACAAG |
| HcMYB1 |  | TCGTCAGTAGCAGCACCAGC | TACAGAGACGAAGCCCAAGATG |
| HcMYB2 |  | TTGCGGCTGGATGGATGAGT | ATTCTCCATCTTAGTCACTTGC |
| HcMYB3 |  | ATGGAGGAGTTGCCTGTAG | CATCGAGAGTTCTGATAGATC |
| HcMYB4 |  | TACTTGGCGTCGTTGGAGTC | ACTTTCTTGGTGCTCTTCGTCG |
| HcMYB5 |  | GGATCACAGATGGGAGATTAC | ATTGGTAGCTCCTCCTACTC |
| HcMYB6 |  | AGCATCCACGACATCACTACG | ACGAATTGAAGAGCAGAGCAGG |
